# Spatio-Temporal Variation Characteristics of PM_2.5_ in the Beijing–Tianjin–Hebei Region, China, from 2013 to 2018

**DOI:** 10.3390/ijerph16214276

**Published:** 2019-11-04

**Authors:** Lili Wang, Qiulin Xiong, Gaofeng Wu, Atul Gautam, Jianfang Jiang, Shuang Liu, Wenji Zhao, Hongliang Guan

**Affiliations:** 1 College of Resource Environment and Tourism, Capital Normal University, Beijing 100048, China; wll_0423@163.com (L.W.); wgf_0307@163.com (G.W.); atul.gautam88@gmail.com (A.G.); 2180902063@cnu.edu.cn (J.J.); 2180902092@cnu.edu.cn (S.L.); 2 Faculty of Geomatics, East China University of Technology, Nanchang 330013, China; xiong_ql@163.com

**Keywords:** PM_2.5_, air pollution, spatio-temporal variation, geographical detector, BTH

## Abstract

Air pollution, including particulate matter (PM_2.5_) pollution, is extremely harmful to the environment as well as human health. The Beijing–Tianjin–Hebei (BTH) Region has experienced heavy PM_2.5_ pollution within China. In this study, a six-year time series (January 2013–December 2018) of PM_2.5_ mass concentration data from 102 air quality monitoring stations were studied to understand the spatio-temporal variation characteristics of the BTH region. The average annual PM_2.5_ mass concentration in the BTH region decreased from 98.9 μg/m^3^ in 2013 to 64.9 μg/m^3^ in 2017. Therefore, China has achieved its *Air Pollution Prevention and Control Plan* goal of reducing the concentration of fine particulate matter in the BTH region by 25% by 2017. The PM_2.5_ pollution in BTH plain areas showed a more significant change than mountains areas, with the highest PM_2.5_ mass concentration in winter and the lowest in summer. The results of spatial autocorrelation and cluster analyses showed that the PM_2.5_ mass concentration in the BTH region from 2013–2018 showed a significant spatial agglomeration, and that spatial distribution characteristics were high in the south and low in the north. Changes in PM_2.5_ mass concentration in the BTH region were affected by both socio-economic factors and meteorological factors. Our results can provide a point of reference for making PM_2.5_ pollution control decisions.

## 1. Introduction

China’s *Air Quality Standards (GB3095-2012)* [1] defines PM_2.5_ as particulate matter with aerodynamic equivalent diameter ≤2.5 μm in ambient air, (also known as fine particulate matter), which is the primary pollutant leading to the decline of urban air quality and frequent haze weather. PM_2.5_ is the most prominent hazard to the human body [2,3,4,5,6]. Previous studies have shown that fine particles, such as PM_2.5_ can enter blood circulation through the alveoli, causing severe harm to the respiratory system [7,8] and cardiovascular system [8], as well as a series of diseases, including carcinogenic illnesses [9], and other health effects such as low birth weight, premature birth, etc. The World Health Organization (WHO) considers the standard for annual mean PM_2.5_ concentration to be 10 µg/m^3^. At present, 92% of the world’s population live in areas where the average PM_2.5_ mass concentration exceeds this value, with approximately 3 million people each year dying from outdoor air pollution [10]. 

As a coping mechanism against air pollution hazards and to protect public health, governments of various countries have issued policies to prevent and control air pollution. In 2009, the United States passed the *Clean Energy and Security Act of the United States of America*, and India passed the *National Green Court Act of 2010* in 2010. In 2012, the Ministry of Environmental Protection of the People’s Republic of China and the General Administration of Quality Supervision, Inspection and Quarantine jointly issued the *Air Quality Standards (GB 3095-2012)*. For the first time, the Chinese government incorporated PM_2.5_ into the primary pollutants monitoring index system. 

Previous studies have focused on the completion of research into PM_2.5_ pollution characteristics [11,12], evolution mechanisms [13], and management and control [14] by using sampling detection [15], high-precision real-time measurements [16,17,18,19], and remote sensing [20,21]. For example, Zhou et al. [22] used PM_2.5_ data retrieved from NASA (National Aeronautics and Space Administration) atmospheric remote sensing images to analyze the temporal and spatial evolution characteristics of PM_2.5_ mass concentrations in China from 2000–2011. It was observed that the distribution trend of PM_2.5_ mass concentration in China was high in the north and east, and low in the south and west, with the highest pollution levels observed in the Beijing–Tianjin–Hebei (BTH) region. Song et al. [23] analyzed air pollution data from more than 1300 national air quality monitoring stations in China. The results of spatial and temporal changes of air pollution in China from 2014–2016 showed that the average PWA (population-weighted-average) concentration in northern China was about 40.4% higher than those in southern China over the past five years. Zhang et al. [24] composed statistics on PM_2.5_ concentrations in 190 Chinese cities from 2014–2015, and found that PM_2.5_ concentrations had significant seasonal variations, with the highest concentration in winter and the lowest concentration in summer. Li et al. [25] showed that PM_2.5_ in the northern BTH region from 2006 to 2013 mainly came from local emissions. In contrast, in the southern and central-southern regions, the meteorological factors were as important as the local emissions of PM_2.5_. Wang et al. [26], based on the monitoring data for 13 cities in the BTH region for 2014, found that the air quality in the northern BTH region was better than those in the middle and southern regions. Serious PM_2.5_ pollution in the BTH region has gradually become a hot research topic in the field of the atmospheric environment [27,28,29,30].

The BTH region is the capital circle of China, and it is also one of the megacity clusters with severe haze across the country. In recent years, under the combined effect of meteorological factors and human factors (intensified air pollution control), the air quality in the area has improved significantly. However, under certain conditions, the outbreak of heavy fog and haze in the BTH region still occurs, which has a serious impact on the health of residents and the harmonious development of the capital circle. In September 2013, the *Air Pollution Prevention and Control Action Plan for Atmospheric Pollution* (hereafter referred to as the *Action Plan*) promulgated by the State Council of China clearly stated that by 2017, the concentration of fine particulate matter in the BTH region, Yangtze River Delta and Pearl River Delta should be reduced to approximately 25%, 20% and 15%, respectively. The average annual concentration of fine particulate matter in Beijing should be controlled to approximately 60 μg/m^3^. The *Action Plan* is thus far the most stringent action plan for the prevention and control of air pollution in China. It is considered an important policy for controlling environmental degradation and improving air quality. In June 2018, the Chinese government issued the *Three-year Action Plan for the Defense of the Blue Sky*, listing PM_2.5_ as the key pollution prevention and control factor, and proposing to focus on the control of air pollution in the BTH region and its surrounding areas. Meanwhile, the number of PM_2.5_ national air-quality monitoring stations increased from 612 in 2013 to 1602 in 2018 [22]. The closing year for the action plan was 2017. So based on the hourly data collected by 102 ground air-quality automatic monitoring stations in the BTH region from 2013 to 2018, this study considered multiple time and regional scales to investigate the trends and effects of PM_2.5_ mass concentration in the BTH region after the implementation of the air pollution control policy. The results can provide a scientific basis for prevention and control policies in the BTH region and surrounding areas. 

## 2. Materials and Methods 

### 2.1. Study Area

**Figure 1:**
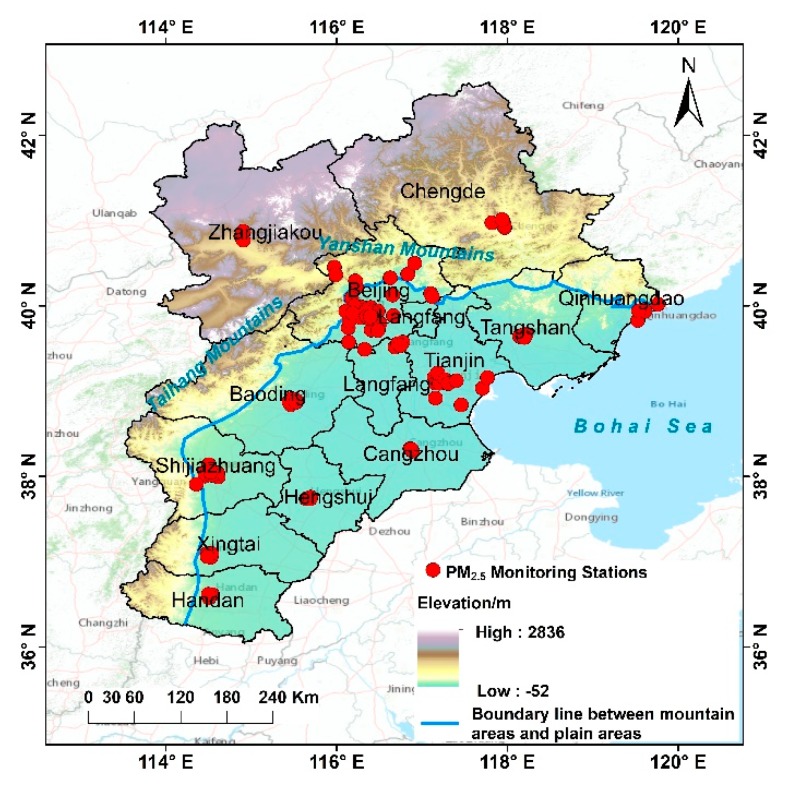
Map of the study area (the mountain areas and plain areas are divided by the blue color line).

The BTH region is surrounded by the Taihang Mountains to the west, the Bohai Sea to the east, Yanshan Mountains to the north, and the North China Plain to the south. The region belongs to the temperate continental monsoon climate, with hot and rainy summers, and cold, dry winters that experience temperature inversion, while the spring and fall seasons are short, windy and rainless. The climate is affected by the structure of the Yanshan–Taihang Mountains, which act as a barrier to the dominant wind direction in the region, and is characterized by terrain that gradually decreases from northwest to southeast. With the frequent occurrence of calm wind and inversion weather, it is not conducive to the diffusion of atmospheric pollutants (Figure 1). The BTH region serves as the core area of the Bohai Rim Economic Circle (Figure S1), and is characterized by high energy consumption, high pollution emissions, and complex air pollution.

### 2.2. Data Control

We acquired PM_2.5_ mass concentration data from a total of 102 monitoring stations distributed throughout the BTH region: 35 in Beijing, 14 in Tianjin, and 53 in Hebei. The data were collected from the Beijing Municipal Environmental Monitoring Center (http://www.bjmemc.com.cn/) [31] and China Air Quality Real-time Distribution Platform (http://106.37.208.233:20035/) [32]. Meteorological data was obtained from the China Meteorological Data Network (http://data.cma.cn/) [33], which included the daily mean data of relative humidity, wind speed and precipitation from 26 meteorological monitoring stations in BTH region. The statistical data of each year used in this study comes from the Beijing Regional Statistical Yearbook [34,35,36,37,38,39], Tianjin Statistical Yearbook [40,41,42,43,44,45], Hebei Economic Yearbook [46,47,48,49,50,51]. Chinese agricultural natural Zoning Data, Chinese land-use remote-sensing monitoring data, Chinese farmland ripening remote sensing monitoring data and Chinese population spatial distribution kilometer grid data were collected from the Chinese Academy of Sciences Resource and Environmental Science Data Center (http://www.resdc.cn/) [52,53,54,55]. What’s more, the cultivated land data comes from Google Earth with a spatial resolution of 0.11 m. The Chinese land-use data is Landsat-8 remote sensing image with a spatial resolution of 1 km. The Chinese farmland ripening data is SPOT-VGT day NDVI, with a spatial resolution of 1 km. The vector Map and DEM (Digital Elevation Model) data of the BTH administrative region were from the National Geomatics Center of China (http://ngcc.sbsm.gov.cn/) [56]. 

The *Air Quality Standards (GB3095-2012)* were jointly issued by the Ministry of Environmental Protection of the People’s Republic of China and the General Administration of Quality Supervision, Inspection and Quarantine in February 2012. It standardizes the classification of environmental air standards, pollutants and concentration limits, monitoring methods, and the effectiveness of data statistics, implementation and supervision (Table S1). According to the *Air Quality Standards (GB3095-2012)*, the missing concentration values and outliers of PM_2.5_ were rejected. 

Because the PM_2.5_ mass concentration data used in this study is hourly data, the average values of 0 to 23 hours of each PM_2.5_ monitoring station were taken as the daily average, and the daily average values of all the PM_2.5_ monitoring stations in each city were taken as the daily average for the city on that day. Furthermore, the daily average values of a calendar month were taken as the monthly average, the daily average values of a calendar season were taken as the seasonal average, and the daily average values of a calendar year were taken as the annual average.

In this study, March, April and May of each year were defined as spring, June, July and August as summer, September, October and November as autumn, and December and the following year’s January and February as winter. According to the quality control code labeled in the Daily Data Set of China Surface Climate Data, the outliers of meteorological data such as relative humidity, wind speed and precipitation were eliminated.

### 2.3. Spatial Autocorrelation

Under the dual influence of spatial interaction and spatial diffusion, geographic data may no longer be independent of each other and will have a specific correlation [57]. Spatial autocorrelation analysis is the most commonly used method of spatial data analysis to study whether the attribute values of different geographical locations are correlated with the attribute values of their adjacent locations and the degree of correlation. The Global Moran’s index (Global Moran’s I) is a commonly used indicator to test global autocorrelation. Therefore, to describe the overall distribution of PM_2.5_ mass concentration in the BTH region, the Global Moran’s I was utilized to explore the spatial difference level of PM_2.5_ mass concentration in the BTH region from 2013 to 2018. The Global Moran’s I is calculated as follows [58]:

(1) $$Moran’s\;I=\frac{n\sum_{i=1}^{n}\sum_{j=1}^{n}W_{ij}(x_{i}-\bar{x})(x_{j}-\bar{x})}{(\sum_{i=1}^{n}\sum_{j=1}^{n}W_{ij})\sum_{i=1}^{n}{\left(x_{i}-\bar{x}\right)}^{2}},(i\neq j)$$

where *n* indicates the total number of cities in the study area;$\;x_{i}$ and $x_{j}$ are the annual concentration of PM_2.5_ in city *i* and city *j*; $W_{ij}$ indicates spatial weight matrix between the city *i* and city *j*, if there is a common edge between *i* and *j*, then $W_{ij}$ = 1, otherwise $W_{ij}$ = 0; $\bar{x}$ is the mean of PM_2.5_ mass concentration. The Global Moran’s I is between −1 and 1. When the Global Moran’s I is positive, it indicates that the PM_2.5_ mass concentration in the study area is spatially agglomerated, and the closer the value is to 1, the more clustered the data is. When the I value is zero, the PM_2.5_ mass concentration in the study area is randomly distributed in space or is not spatially correlated. If the value of I is less than zero, it indicates that the mass concentration of PM_2.5_ has a negative spatial correlation, and the value of I tends to –1, which indicates that the spatial dispersion of PM_2.5_ is stronger.

### 2.4. K-Mean Clustering Algorithm

Clustering analysis is a method of clustering similar values and forming multiple clusters; the similarity of the values within a cluster is high and the difference between values in different clusters is high. The k-mean algorithm is an iterative clustering analysis algorithm. It selects k objects randomly to be part of the initial clustering center. It then calculates the distance between each object and each clustering center and assigns each object to the nearest clustering center. A clustering center and the objects assigned to it together represent a cluster [59]. The calculation formula of the cluster center is as follows:

(2) $$C_{k}=\frac{1}{\left|C_{k}\right|}\underset{x_{i\in C_{k}}}{\sum }x_{i}$$

where $C_{k}$ indicates the center of the k-th cluster. $\left|C_{k}\right|$ indicates the number of data objects in the k-th cluster. 

### 2.5. Geographic Detector 

The geographic detector is a set of statistical methods for detecting spatial heterogeneity and revealing the driving factors behind it [22,60]. The model used its factor detector to determine whether factor X is the reason for driving the spatial differentiation of variable Y [22]. In addition, the geographic detector can also identify the direct interaction of the different factors of X_s_, that is, whether the interaction between factor X_1_ and factor X_2_ will increase or decrease the explanatory power of the variable Y. Because it can describe the cause and mechanism of spatial patterns of geographical elements by detecting both numerical data and qualitative data, it has been gradually applied to public health [61,62,63,64], social economy [65,66,67,68], and ecological environment [69,70,71,72]. The principle of geographic detector is shown in Figure S2, and the geographic detector model is defined as follows:

(3) $$q=1-\frac{\sum_{h=1}^{L}N_{h}\sigma_{h}^{2}}{N\sigma^{2}}$$

where q indicates the explanatory power of the influencing factor of variable Y; *h* = 1… *L* is the second region of variable Y or factor X; $N_{h}$ and *N* are the number of sample points in the region *h* and the number of sample points in the whole region, respectively. What’s more, *N = N*_1_ *+ N*_2_ *+ … + N_h_*. $\sigma_{h}^{2}\;$and$\;\sigma^{2}$ are the variances of the secondary region *h* and the whole region variable Y, respectively. The value range of *q* is [0, 1]. The larger the value of *q*, the stronger the explanatory power of factor X to variable Y, and vice versa. In certain extreme cases, the value of *q* is 0, indicating that factor X is independent of variable Y. The value of *q* is 1, indicating that factor X completely controls the spatial distribution of Y. 

## 3. Results

### 3.1. Temporal Variations

#### 3.1.1. Overview of Particulate Matter (PM_2.5_) Pollution 

From 2013–2018 the average annual PM_2.5_ mass concentration in the BTH region decreased from 98.9 μg/m^3^ in 2013 to 55.6 μg/m^3^ in 2018, with an average annual decrease of 8.7 μg/m^3^, and a decrease of 43.8% in the six-year period (Table 1). China has achieved its goal of reducing the concentration of fine particulate matter in the BTH region by 25% by 2017. The average mass concentration of PM_2.5_ was larger than the median, showing a positive skewness. PM_2.5_ mass concentration declined year by year, and the number of cities whose PM_2.5_ mass concentration reached the annual average limit of the grade II standard (35 μg/m^3^) gradually increased. In 2015 and 2016, the average PM_2.5_ mass concentration of only one city (Zhangjiakou) met the standard, while in 2017 and 2018, the average PM_2.5_ mass concentration of two cities (Zhangjiakou and Chengde) met the standard. The number of days with excellent and good air quality increased from 13% and 33% in 2013 to 33% and 47% in 2018 respectively, while the proportion of severely polluted days decreased year by year. By 2018, the proportion of severely polluted days had been zero. The occurrence of good weather has increased significantly and air pollution control actions have achieved remarkable results.

**Figure 2:**
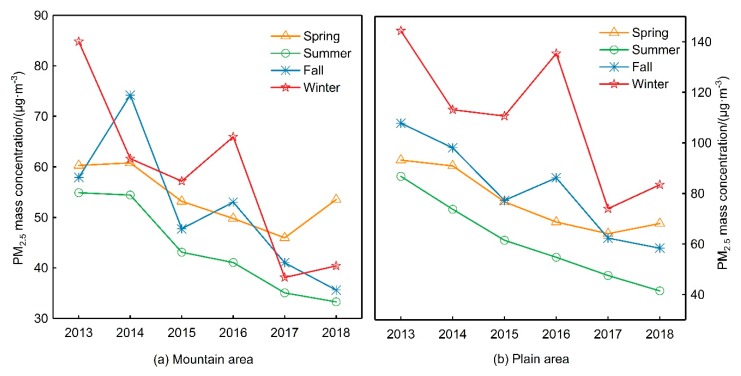
Seasonal variation of PM_2.5_ mass concentrations of BTH from 2013–2018.

The PM_2.5_ mass concentration in the mountain areas of the BTH region was higher in spring, fall and winter than that in summer. PM_2.5_ mass concentration decreased year by year in summer, while there was a large fluctuation range in the other three seasons. The PM_2.5_ mass concentration in plain areas showed significant seasonal variation (Figure 2). From 2013–2016, the PM_2.5_ mass concentration order was winter > fall > spring > summer. From 2017–2018, the PM_2.5_ mass concentration order was winter > spring > fall > summer. Additionally, the PM_2.5_ mass concentration in the plain areas was higher than that in mountainous areas in all seasons. From 2013 to 2015, PM_2.5_ mass concentration decreased sharply in winter in plain and mountainous areas but increased in the winter of 2016. Winter is the heating period in the BTH region. Whether in mountainous or plain areas, industrial enterprises and residents use coal as a fuel for heating. China began to control the total consumption of coal in 2013 and increased the intensity of non-fossil energy utilization to replace coal. Thus, from 2013 to 2015, PM_2.5_ mass concentration in winter in the BTH region dropped sharply. With the release of the non-capital function of Beijing in 2015 and the promotion of the coordinated development of the BTH region, the construction of urbanization in the BTH region accelerated in 2016. Therefore, large-scale construction sites are exposed in winter, and dust from the exposed ground can aggravate PM_2.5_ pollution. There was little seasonal difference in the PM_2.5_ mass concentration in both the mountainous and plain areas in 2017, with the PM_2.5_ mass concentration decreasing significantly in winter. This is due to the implementation of the *Air Pollution Comprehensive Control Action Plan for air pollution prevention and control in the BTH region and surrounding areas* (referred to as the *key plan*) in September 2017, which effectively controlled the PM_2.5_ pollution in the BTH region in winter. Atmospheric pollutants produced by loose coal combustion and those discharged by high energy-consuming enterprises are important sources of air pollution in the heating season in the BTH region and surrounding areas. The *key plan* proposes to use clean energy (electricity and natural gas) instead of coal to reduce the pollutants emitted by coal combustion and implement the staggered peak production program for high-emission enterprises in the areas surrounding the BTH region.

**Figure 3:**
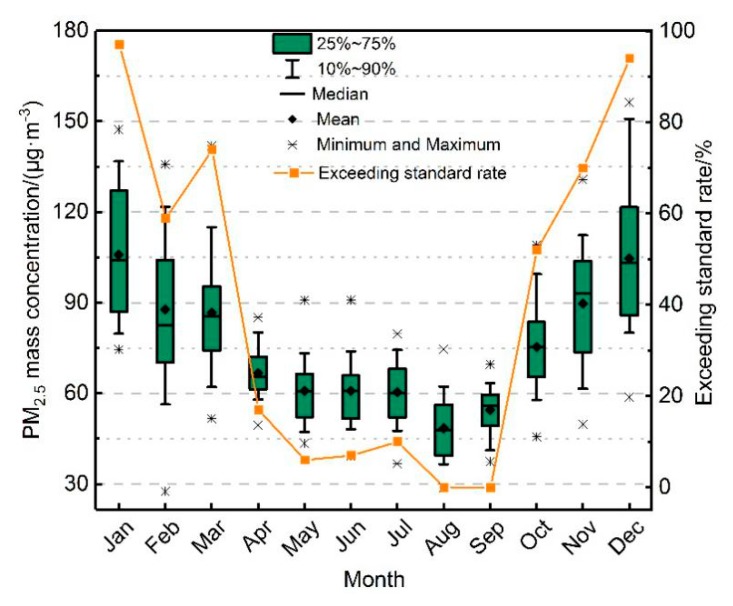
Box-plot of monthly PM_2.5_ mass concentration of BTH from 2013–2018.The proportion of days on which the PM_2.5_ concentration exceeded 75 μg/m^3^ per month to the total number of days per month is the exceeding rate.

The monthly average PM_2.5_ mass concentration in the BTH region from 2013–2018 showed a “U-shaped” pattern, which declined from January to March (Figure 3). The PM_2.5_ mass concentration from April to September was low, and the PM_2.5_ mass concentration increased significantly from October to December. The 24-hour average limit of the grade II standard (75 μg/m^3^) for PM_2.5_ in the *Air Quality Standards* was used to evaluate the exceeding rate (Table S1). The change of the daily exceeding rate of PM_2.5_ mass concentration was similar to the PM_2.5_ daily average mass concentration. The average mass concentration of PM_2.5_ from January to March was 93.5 μg/m^3^, and the daily average exceeding standard rate was approximately 59%–97%. From April to September, the mass concentration of PM_2.5_ ranged from 48.6 to 66.7 μg/m^3^, and the daily average exceeding rate was lower than 20%. The daily average exceeding rate from August and September was zero, and the excellent and good weather mostly occurred from April to September. From October to December, PM_2.5_ ranged from 75.4 to 104.6 μg/m^3^, and the daily average exceeding rate was 52%–94%. PM_2.5_ pollution was highest in January (105.9 μg/m^3^) and December (104.6 μg/m^3^), and lowest in August (48.6 μg/m^3^) and September (54.5 μg/m^3^). This is because January, November and December are heating periods in the BTH region, and the atmospheric pollutants from coal-burning are not easy to diffuse in winter.

#### 3.1.2. PM_2.5_ Mass Concentration in Regions and Cities 

**Figure 4:**
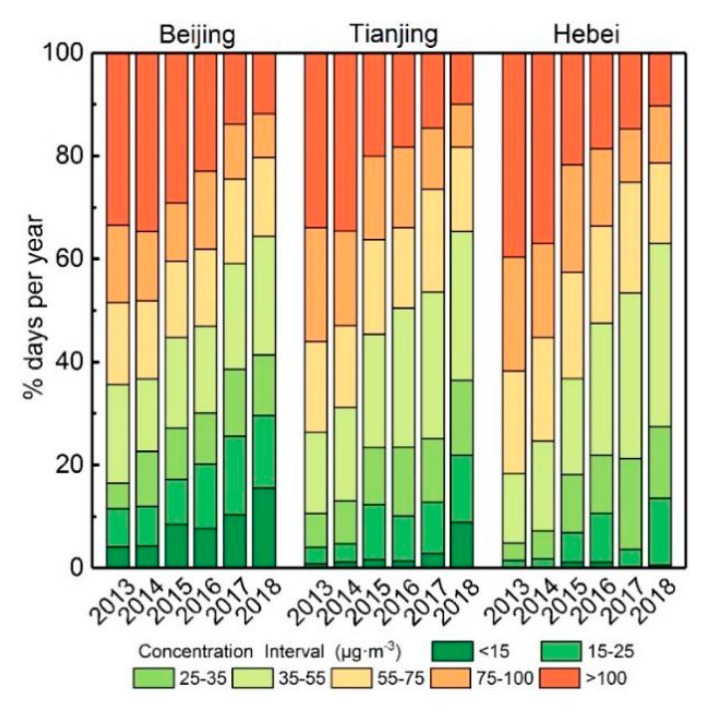
Annual range of daily PM_2.5_ mass concentration for Beijing, Tianjin, Hebei from 2013–2018.

According to 24-hour average limits of the grade I and grade II standards for the limit of annual mass concentration of PM_2.5_ in environmental air quality standards, the annual average mass concentration of PM_2.5_ is divided into seven levels. From the regional perspective, the proportion of days in each concentration range from 2013–2018 in Beijing, Tianjin and Hebei province was studied (Figure 4). The variation of trends in PM_2.5_ mass concentration ranges in the three regions was similar. Days in which PM_2.5_ mass concentration was below 35 μg/m^3^ (the 24-hour average grade I standard) increased year by year, while the days in which mass concentration was over 75 μg/m^3^ (the 24-hour average grade II standard) decreased year by year. The days when PM_2.5_ mass concentration was less than 35 μg/m^3^ in Beijing, Tianjin and Hebei increased from 16.4%, 10.6% and 4.9% in 2013 to 41.4%, 36.4% and 27.5% in 2018, respectively. Dense polluted areas with average annual mass concentrations of PM_2.5_ exceeding 100 μg/m^3^ in Beijing appeared in 2013–2015, while heavy polluted areas in Tianjin and Hebei Province appeared in 2013–2014. The proportion of days exceeding 100 μg/m^3^ in each region was less than 15% after 2017. The results show that the low-pollution areas with PM_2.5_ mass concentration lower than 35 μg/m^3^ in Beijing, Tianjin and Hebei Province are increasing year by year, the heavy-pollution areas with PM_2.5_ mass concentration higher than 100 μg/m^3^ are decreasing continuously.

The reason for PM_2.5_ pollution decreasing year by year in the three regions is that the three regions have carried out high energy consumption industrial control actions. In 2014, the Beijing government issued regulations on the prevention and control of air pollution in the municipality. The regulations stipulate that the coverage rate should be increased, and facilities for burning coal, heavy oil and residual oil should be prohibited. The proportion of PM_2.5_ daily average concentration of less than 35 μg/m^3^ has increased since 2015 (Figure 4). In 2015, the regulations on prevention and control of atmospheric pollution were proposed to eliminate high energy-consuming industries, close small power plants, replace coal-fired power plants with clean energy, and replace coal with natural gas. At the same time, it is forbidden to incinerate straw, foliage and other substances which produce smoke and dust in the open air. In 2016, the provincial government carried out the transformation of highly polluting fuel facilities. In 2015, the Tianjin Air Pollution Prevention and Control Regulations proposed the elimination of high-energy-consuming industries, the closure of small power plants, the replacement of coal-fired power plants with clean energy, and the replacement of coal with natural gas by boiler fuel. Furthermore, it is proposed to ban the burning of straw, leaves and other substances that produce soot. In 2016, the Hebei Provincial Government carried out the transformation of highly polluting fuel facilities. Therefore, under the joint development of a several of air pollution control policies, the proportion of PM_2.5_ heavy pollution days in the BTH region has been declining year by year.

**Figure 5:**
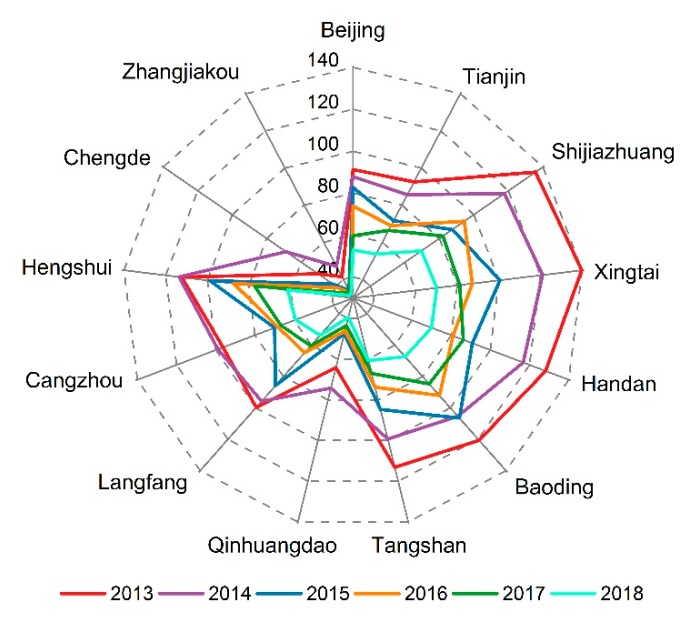
Annual variation of PM_2.5_ mass concentration in different cities in BTH from 2013–2018. The numbers represent the PM_2.5_ mass concentrations in μg/m^3^.

In 2013, the average annual PM_2.5_ mass concentration in 13 cities in BTH region was higher than 35 μg/m^3^ (Figure 5). Among them, PM_2.5_ pollution of Xingtai was the most serious, where the annual mass concentration reached as high as 139.8 μg/m^3^, exceeding the annual average grade II standard of 104.8 μg/m^3^. The average annual concentration of PM_2.5_ in Zhangjiakou was below 35 μg/m^3^ between 2015 and 2018. The average mass concentration of PM_2.5_ in Baoding, Shijiazhuang, Xingtai, Hengshui and Handan in 2013–2014 exceeded 100 μg/m^3^. Moreover, the decline rate of PM_2.5_ mass concentration in these five cities was the highest during the study period. After five years of air pollution control in 13 cities, the air quality has improved.

Baoding, Shijiazhuang, Xingtai, Hengshui, and Handan are five cities where heavy industry is concentrated and most energy is coal-fired, so air pollution is relatively severe compared with other cities. In 2013, the air pollution prevention and control action plan proposed to strengthen comprehensive control of air pollution in industrial enterprises, control the total consumption of coal, increase the supply of natural gas, increase the intensity of non-fossil energy use and other measures to replace coal. In 2017, the BTH region began to step up efforts to reduce excess capacity, and by the end of October 2017, it had closed down illegal "small, scattered and polluting" enterprises. At the same time, Beijing, Tianjin, Langfang, and Baoding completed the construction of “coal-prohibited areas”, realizing clean energy heating in winter. Air quality in cities with heavy PM_2.5_ pollution has improved as a result of strict pollution control by industrial enterprises. However, in 2018, except in Zhangjiakou and Chengde, the average annual PM_2.5_ concentration in the cities remained over 35μg/m^3^. Thus, it is evident that air pollution control actions should continue.

### 3.2. Spatial Variations

#### 3.2.1. PM_2.5_ Spatial Evolution 

**Figure 6:**
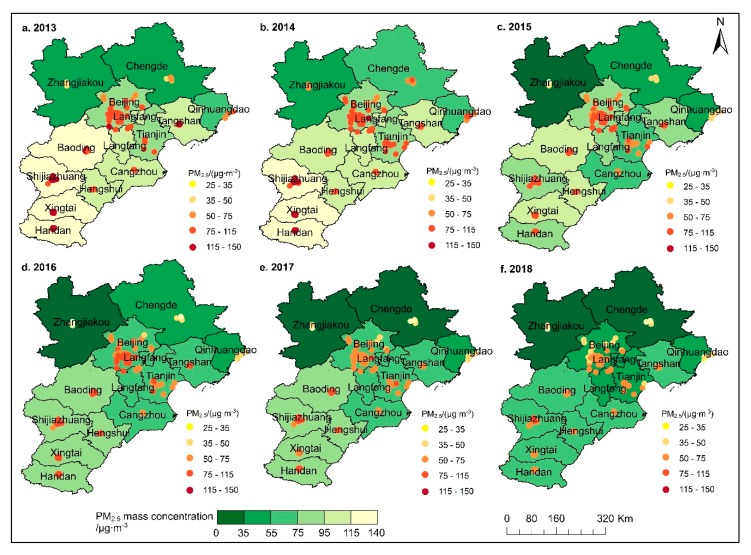
Spatial characteristics of annual PM_2.5_ mass concentration of BTH from 2013–2018.

According to the annual average PM_2.5_ limit set by China’s *Air Quality Standards (GB3095-2012),* and by using natural breaks (Jenks) method in ArcGIS, PM_2.5_ pollution in the study area is of three types: good (PM_2.5_ value is in the range (0, 35)), lightly polluted (PM_2.5_ value is in the range (35, 75)), and heavily polluted (PM_2.5_ value is in the range (75, 150)). The natural breaks (Jenks) method classifies the research units with similar attribute values according to different classification principles, and divides them into several types according to the data indicators. PM_2.5_ pollution in the BTH region showed a low spatial distribution pattern in the northwest mountainous area and a high spatial distribution pattern in the southeast plain over the six-year study period (Figure 6). The trend of PM_2.5_ pollution was distributed regionally and the range of heavy pollution concentration was shown to be shrinking year by year. PM_2.5_ pollution in various cities was also shown to be decreasing. The difference of PM_2.5_ mass concentration between different cities is decreasing year by year, and the pollution of PM_2.5_ has been reduced significantly in six years. The BTH region witnessed severe PM_2.5_ pollution in 2013, which was reduced by 2018. The difference in PM_2.5_ mass concentration from 2013–2018 was 43.3 μg/m^3^. Also, the proportion of PM_2.5_ monitoring stations with annual average PM_2.5_ mass concentrations below 35 µg/m^3^ continued to increase from 0 in 2013 to 9% in 2018 (Table S2). Before 2016, the mass concentration of PM_2.5_ in most stations was between (75,150). After 2016, the mass concentration of PM_2.5_ in most stations was between (35, 75). In 2013, the proportion of sites with mass concentrations between (75,150) was as high as 80%, while in 2018 it dropped to 3%. Furthermore, the proportion of sites with mass concentrations between (35, 75) increased from 20% in 2013 to 88% in 2018. Regardless of the year, the stations with lower PM_2.5_ mass concentrations were located in Zhangjiakou and Chengde in the northern part of the BTH region and differences in the spatial distribution of PM_2.5_ decreased every year (Figure 6).

The central and southern cities of the BTH region witnessed the most severe PM_2.5_ pollution from 2013–2015. These cities experienced long-term high pollution compared with northern cities. From 2016–2018, the difference in PM_2.5_ pollution between the north and the south was gradually weakening. From 2013 to 2015, Xingtai was the highest pollution center of PM_2.5_ in the BTH region, and the pollution degree decreased from Xingtai to three neighboring cities. From 2016 to 2017, the number of stations and cities that met the annual average grade II standard (35 μg/m^3^) increased (Table S2). In 2018, the air quality in the northern parts of the BTH region was generally good. The PM_2.5_ pollution in the central and southern cities of the BTH region was distributed continuously. Thus, future air pollution control action in the BTH region should focus on the central and southern urban agglomerations.

#### 3.2.2. Spatial Autocorrelation Analysis

Moran’s I in the BTH region was positive for the six-year study period, indicating that the average PM_2.5_ mass concentration in 13 cities showed spatial agglomeration, and as time went on, the spatial agglomeration level showed a trend of first rising and then declining (Table 2). The Global Moran’s I reached its maximum in 2015 (Moran’s I = 0.8), indicating that 2015 was the peak of PM_2.5_ pollution spatial agglomeration in the BTH region. 

**Figure 7:**
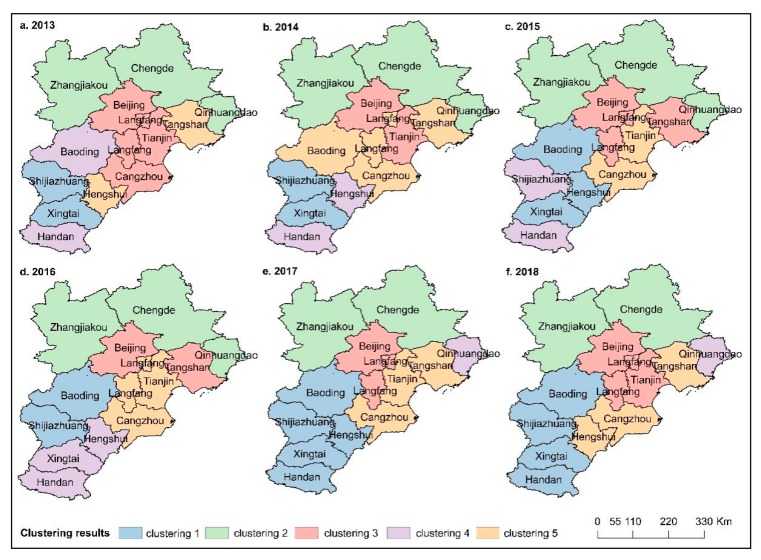
The clustering distribution of PM_2.5_ for BTH from 2013–2018. Each cluster is denoted by a unique color.

In order to further explore the spatial agglomeration of PM_2.5_ pollution, the K-mean algorithm was used to analyze the PM_2.5_ mass concentration in 13 cities in the BTH region. The clustering results show that the spatial distribution of PM_2.5_ pollution in the BTH region is in agreement with the previous results (Figure 7) [73]. Combined with Figure 6, we know that Zhangjiakou, Chengde and Qinhuangdao in the northern part of the Yanshan–Taihang Mountains were low-pollution areas of PM_2.5_, while the pollution of PM_2.5_ in Shijiazhuang, Baoding, Xingtai and Handan were more serious. The northern part of the BTH region had higher terrain, while most of the heavily polluting heavy industries were located in the central and southern cities [74]. The spatial distribution characteristics of PM_2.5_ pollution in the BTH region are closely related to topography and industrial production structure.

### 3.3. Driving Forces of PM_2.5_ Pollution

Previous studies have found that large-scale PM_2.5_ spatial change is mainly affected by global climate change, population density, land use, and economy [75,76,77,78]. However, the spatial scale of the BTH region is relatively small. Thus, in this study, the PM_2.5_ mass concentration was selected as variable Y, and sown area of farm crops (*X*_1_), urban greening rate (*X*_2_), GDP (gross domestic product) (*X*_3_), gross domestic product of secondary industry (*X*_4_), completed floor space (*X*_5_), population density (*X*_6_), car ownership (*X*_7_), average wind speed (*X*_8_), relative humidity (*X*_9_), and precipitation (*X*_10_) were selected as factor X. The driving effect of each index on the PM_2.5_ mass concentration in the BTH region was determined by geographic detector. The interpretation of the detection indices are shown in Table S3. Since China’s social and economic data for 2018 has not been announced yet, the research on the factors affecting PM_2.5_ in the BTH region is only from 2013–2017.

The detection results showed that the change of PM_2.5_ mass concentration in the BTH region was closely related to socio-economic and natural factors (Table 3). Among them, the sown area of farm crops (*X*_1_), urban greening rate (*X*_2_), completed floor space (*X*_5_) and population density (*X*_6_) had a significant impact on the PM_2.5_ mass concentration. In 2013, 2014, 2016 and 2017, the detection force q of the sown area of farm crops(*X*_1_) were 0.6, 0.6, 0.4 and 0.5 respectively, showing inverted U-shaped change characteristics. From 2013–2017, the detection force q of completed floor space (*X*_5_) and population density (*X*_6_) to PM_2.5_ were always high and stable. In 2013 and 2015, the main driving forces of PM_2.5_ pollution change were sown area of farm crops (*X*_1_), urban greening rate (*X*_2_), completed floor space (*X*_5_) and population density (*X*_6_). However, the main driving factors of PM_2.5_ mass concentration change in 2017 were urban greening rate (*X*_2_), completed floor space (*X*_5_), population density (*X*_6_) and precipitation (*X*_10_). In 2013, 2015 and 2016, the impact of human activities on PM_2.5_ is greater than that of natural factors. The detection force q values for which the two-factor interaction is greater than the effect of a single factor on PM_2.5_ are highlighted in bold in Table S4. The influence of socio-economic factors on PM_2.5_ is more stable than that of natural factors on PM_2.5_. 

The effect of the crop sowing area (*X*_1_) on PM_2.5_ mass concentration in the BTH region was the smallest in 2016. The North China Plain in the BTH region is the main winter wheat and maize production area in China (Figure S3, Figure S4). It is also a serious area of straw burning. Crop growth can inhibit PM_2.5_ emission from bare cultivated land. However the crop harvest season (June and October) is the peak period of straw burning. Straw burning is one of the important causes of pollution processes such as heavy fog and haze in autumn [79]. Although Beijing, Tianjin, and Hebei respectively formulated policies for prohibiting outdoor burning of straw in 2014, 2013 and 2015, straw burning still existed (Table S5). In 2015, the incineration of crop straws in Hebei was about 2 × 10^6^ t, the total black carbon (BC) emissions of Beijing, Tianjin and Hebei was 15.8 t, 53 t and 10^3^ t, respectively [73]. Black carbon usually refers to the amorphous carbon emitted from incomplete combustion of biomass and fossil fuels. It is an important component of PM_2.5_ [80]. The crop sowing area can indirectly represent straw incineration, so the changes in the crop sowing area will affect PM_2.5_ mass concentration [22]. Urban greening (*X*_2_) covers the exposed land, thereby suppressing ground dust. In addition, secretions such as villi and mucus on the surface of plant leaves can adsorb fine particles, thereby reducing PM_2.5_ pollution. From 2013 to 2016, the detection force q value of the gross domestic product of secondary industry (*X*_4_) to PM_2.5_ is higher than that of GDP (*X*_3_) to PM_2.5_. The second industries include mining and manufacturing industries, while the Southern Hebei urban agglomerations (Langfang, Baoding, Shijiazhuang, Cangzhou, Hengshui, Xingtai, Handan) is an area of heavy industry in the BTH region, and the gross industrial output contributes a lot to the regional economy. Meanwhile, the impact of the gross domestic product of secondary industry (*X*_4_) on PM_2.5_ is higher than that of GDP (*X*_3_) on PM_2.5_ pollution. In 2017, the industrial enterprises in the BTH region implemented the peak load transport system for bulk materials. The steel production capacity of the heavily polluted cities such as Shijiazhuang, Tangshan, and Handan was limited by 50% in the heating season. Therefore, the impact of the gross domestic product of secondary industry (*X*_4_) on PM_2.5_ in 2017 was lower than that of GDP (*X*_3_) on PM_2.5_ pollution. Car ownership (*X*_7_) had little influence on the change of PM_2.5_ mass concentration. The detection force q of PM_2.5_ is relatively stable for population density (*X*_6_) and completed floor space (*X*_5_). The population of the BTH region is mainly concentrated in the Plain Area (Figure S5). The increase in population density will result in an increase in resource consumption and housing demand. At the same time, with the rapid expansion of the urban area and the high-intensity urban transformation, the dust generated during the construction process has gradually become one of the main sources of urban atmospheric particulates. In 2013, 2015 and 2017, PM_2.5_ emissions from bare construction land in Beijing were 3.1 × 10^4^ t, 2.4 × 10^4^ t and 4 × 10^4^ t [81]. The emission of construction dust in 2013 in Tianjin was 2.9 × 10^4^ t. It can be seen that the change of completed floor space (*X*_5_) will affect the surface PM_2.5_ concentration. The driving force of average wind speed (*X*_8_) on the change of PM_2.5_ mass concentration gradually weakened. The detection force q value decreased from 0.3 in 2013 to 0.01 in 2017. The weather system in the BTH region is relatively stable because of the small area and the backing of the Yanshan–Taihang Mountains (Figure 1). In 2013–2017, the PM_2.5_ mass concentration in the BTH region decreased year by year, and the difference of PM_2.5_ mass concentration among cities was narrowed. When the average wind speed is relatively stable, the influence of wind speed on the spatial distribution of PM_2.5_ decreases. The detection force q of precipitation (*X*_10_) on PM_2.5_ was relatively low in 2016 (q = 0.01), because compared with previous years, precipitation in heavily polluted PM_2.5_ areas in Shijiazhuang, Xingtai and Handan was abundant, while precipitation in other heavily polluted PM_2.5_ areas such as Baoding, Hengshui and Langfang was still less, so the influence of precipitation (*X*_10_) on PM_2.5_ spatial distribution was reduced. The detection power q of PM_2.5_ mass concentration was relatively stable for the sown area of farm crops (*X*_1_), GDP (*X*_3_), gross domestic product of secondary industry (*X*_4_), completed floor space (*X*_5_), population density (*X*_6_) and relative humidity (*X*_9_), while the driving force q of average wind speed (*X*_8_) and precipitation (*X*_10_) for the change of PM_2.5_ mass concentration fluctuated greatly. 

## 4. Discussion

We collected six years of PM_2.5_ mass concentration data for the BTH region (from January 2013 to December 2018) and studied the spatio-temporal variation characteristics of fine particulate matter in the BTH region before and after the implementation of the air pollution prevention and control action plan. From 2013–2018, PM_2.5_ pollution in the BTH region was reduced to a great extent. Compared with 2013, the average mass concentration of PM_2.5_ decreased by 43.3 μg/m^3^ in 2018, and the number of cities and stations meeting the limit of grade II standard of PM_2.5_ in China’s *Environmental Air Quality Standard* increased year by year. There were seasonal variations in the BTH region plain areas. PM_2.5_ mass concentration in winter was the highest, and in summer it was the lowest. Changes in monthly average PM_2.5_ mass concentration showed a U-shaped pattern with the highest values in January and December, lowest in August and September. During the heating period of the BTH region in winter, the atmospheric pollutants discharged from boilers after burning coal increase significantly, while the atmosphere is relatively stable in winter. The frequency and intensity of inversion are higher and the duration of inversion is longer. This climate condition increased the difficulty of pollutant diffusion and dilution [82]. The BTH region has a dry spring climate with more wind and less rain, so the PM_2.5_ mass concentration was higher than that in summer, and the temperature rises, atmospheric stability decreases and rainfall is more concentrated in summer. In addition, the dust brought by Inner Mongolia high pressure will aggravate PM_2.5_ pollution in spring [83]. All these climatic conditions are conducive to the diffusion, settlement and dilution of air pollutants [84]. The mass concentration of PM_2.5_ in the BTH region was 64.9 μg/m^3^ in 2017, 34% lower than that in 2013. The average number of days with heavy air pollution in 13 cities in the BTH region decreased significantly, and the regional air quality continued to show an overall improvement trend. The variation of trends in PM_2.5_ mass concentration ranges in the three regions (Beijing, Tianjin, Hebei Province) was similar. Legislation and policy played a crucial role in air pollution management [85,86,87]. Table S5 lists the legislations and policies on air quality protection that were adopted by China. Chinese government enacted the *Air Pollution Prevention and Control Action Plan* in 2013. At the same time, the regional coordination mechanism of the BTH region was established. After that, the *Environmental Protection Law* and *Air Pollution Prevention and Control Law* were enacted by the Chinese government in 2015 (Table S5). To implement the Action Plan, Beijing put forward the *Clean Air Action Plan for 2013–2017* in 2013. Its goal was to control the fine particulate matter levels in Beijing to about 60 μg/m^3^ by 2017. The mass concentration of PM_2.5_ in Beijing was 59.7 μg/m^3^ in 2017, 31.6 μg/m^3^ lower than that in 2013 (91.3 μg/m^3^), in line with the *Clean Air Action Plan for 2013–2017* target. The incidence of good weather has increased significantly, and the prevention and control of air pollution in the BTH region has achieved remarkable results.

In terms of spatial distribution, PM_2.5_ mass concentration in southeast plain areas is higher than that of the northwest mountainous areas. The central and southern cities of the BTH region have large population density (Figure S5), large energy consumption, and a large number of high-pollution, high-consumption, high-emission enterprises, and the consumption of fossil fuels is large [88,89], which has caused the central and southern cities to become PM_2.5_ high-pollution areas. The spatial distribution characteristics of PM_2.5_ pollution in the BTH region are closely related to industrial production structure. Furthermore, the topographic and climatic factors also have great influence [90]. The overall topographic characteristics of the BTH region are high in the northwest and low in the southeast, while Zhangjiakou and Chengde are located in mountainous areas, which are much higher than those in the southern plain areas. When southeast winds prevailed, due to the obstruction of the Yanshan–Taihang Mountains, atmospheric pollutants accumulated over the central and southern cities, and were not easy to diffuse. When northwest wind prevailed, the cities of the central and southern BTH region were in the downwind direction of PM_2.5_ pollution diffusion, which led to a sharp contrast between PM_2.5_ high-pollution areas such as Baoding, Shijiazhuang, Xingtai and Handan, and PM_2.5_ low-pollution areas such as Zhangjiakou and Chengde. The most polluted areas are Baoding, Shijiazhuang, Xingtai, Hengshui and Handan. 

Table 3 shows how the sown area of farm crops (*X*_1_), urban greening area (*X*_2_), completed floor space (*X*_5_) and population density (*X*_6_) had a greater impact on the PM_2.5_ mass concentration compared with other indicators. The BTH region consists of four natural agricultural areas (Figure S4a). Dry land mainly covers the plain area; a small amount of dry land also occupies the mountainous areas (Figure S4b). Most crops in the northern mountainous areas and eastern plain areas of the BTH region are ripe once a year, and in the southern areas, they are ripe thrice or twice a year (Figure S4c). Summer (June–August) large-scale crop growth can effectively inhibit PM_2.5_ emissions from bare cultivated land (Figure S3). In spring (March–May) and autumn (September–November), the phenomenon of straw burning is more serious in northern China. Heavy pollution caused by straw burning mainly occurs in autumn [91]. Previous study showed that biomass combustion is an important global source of air pollutant emissions [92,93]. The North China Plain agricultural area in the BTH region is the largest winter wheat production area in China. Large-scale straw burning can cause serious air pollution accidents in this area [94,95]. In winter (December and following year’s January and February), plants lie dormant and the cultivated land is bare (Figure S3), drought and gale weather occur frequently, and surface wind erosion increase in intensity [96]. Previous studies have shown that fugitive dust emission is one of the main contributing sources to atmospheric particulate matter in northern cities of China [97]. The contribution of straw incineration to atmospheric particulate matter and the amount of dust emitted from bare cultivated land can be assessed by the change of crop area. In recent years, with the relaxation of Beijing’s non-capital functions and the acceleration of urban expansion, a large number of construction sites have emerged in the city, whose dust also directly affects the PM_2.5_ pollution in the BTH region. Urban greening can suppress dust on the ground. Meanwhile, leaves of plants can retain and suppress some dust, and PM_2.5_ pollution can also be reduced. However, the driving factors of PM_2.5_ pollution change in the BTH region are complex. In addition to natural factors such as topography, meteorology, industrial pollution, motor vehicle exhaust emissions, construction dust, biomass combustion and other human factors have a more significant impact on PM_2.5_ pollution. 

At present, there are many studies on the temporal and spatial distribution of PM_2.5_ in the BTH region, but most of them only analyze the temporal and spatial distribution of PM_2.5_ in a single year or a short time series [26,30,84,95,96,97,98,99,100,101,102] or only analyze the temporal and spatial distribution of PM_2.5_ without discussing its causes [103,104,105]. The conclusions of the analysis of PM_2.5_ spatial distribution pattern revealed in this paper are in line with previous research conclusions, and can more systematically reflect the changes of PM_2.5_ pollution in different time scales in the BTH region in a longer time series. The terrain of the BTH region is high in the northwest and low in the southeast. Most of the heavily polluting enterprises are located in the central and southern plains. The difference between the PM_2.5_ mass concentration in the northern mountainous area and the PM_2.5_ mass concentration in the plain area is large, so this paper also discusses the PM_2.5_ mass concentration changes in the BTH region mountainous area and the plain area, and uses the geographic detector to explore the factors affecting PM_2.5_ in the BTH region. By comparing the changes of PM_2.5_ mass concentration in the BTH region for the past six years, this paper finds that the period and region where PM_2.5_ mass concentration is greatly reduced is closely related to the air pollution control policies and laws in the BTH region in recent years. However, the impact of other factors outside the air pollution control policy on PM_2.5_ concentration changes is not excluded. Due to the large gap between the energy structure and the consumption structure of the industrial structure of each city, the key factors leading to the change of PM_2.5_ pollution are also different. At present, the BTH region is in the critical transition period of industrial restructuring and green development. Therefore, it is necessary to explore the long-term PM_2.5_ pollution change of PM_2.5_ under strict policy supervision.

There are still some limitations in this study. For example, the population-density data and car-ownership data used by geographic detectors need to be further refined, rather than stay at the provincial level.

## 5. Conclusions

In this study, we analyzed the spatio-temporal variation characteristics of PM_2.5_ in the BTH region from 2013–2018, tested the results of air pollution prevention and control actions, and further explored the driving force of PM_2.5_ pollution. Our main conclusions are as follows. PM_2.5_ mass concentration declined gradually in the BTH region—particularly in the severely polluted areas (Xingtai, Shijiazhuang, Baoding, Handan)—during the study period. A strong seasonal trend was observed in BTH plain areas, with highest in winter and the lowest in summer. The PM_2.5_ pollution showed a spatial distribution pattern of high in the south and low in the north, higher PM_2.5_ pollution was mainly found in Xingtai, Shijiazhuang and Baoding. Moran’s I in BTH region was positive in the study period, indicating that PM_2.5_ pollution in 13 cities showed spatial agglomeration. Furthermore, socio-economic factors and meteorological factors have an impact on the spatio-temporal patterns of PM_2.5_ mass concentration in BTH region. With the optimization of the regional coordination mechanism for the BTH region and more stringent laws such as the *Air Pollution Prevention and Control Action Plan* in 2013, the PM_2.5_ pollution in the BTH region has significantly reduced. In 2018, the PM_2.5_ concentration in the BTH region was 55.6 μg/m^3^, which exceeded the annual average grade II standard (35 μg/m^3^). The air pollution remains serious and air pollution control should continue.

Our study contributes to the field as it increases understanding of the status and variations of PM_2.5_ mass concentration in the BTH region. We also expect to provide some valuable references to the other countries around the world when facing regional air pollution problems.

## Figures and Tables

**Table 1:** Summary of daily particulate matter (PM_2.5_) mass concentration in the Beijing–Tianjin–Hebei (BTH) region.

Statistic	2013	2014	2015	2016	2017	2018
PM_2.5_ mass concentration (μg/m^3^)						
Annual Average	98.9	94.8	77.1	69.9	64.9	55.6
Minimum	8.8	13.5	10.1	10.5	10.5	8.9
Median	81.6	78.7	62.1	58.2	52.9	44.7
Maximum	326.3	290.1	313.3	305.4	260.3	244.9
Std. Dev.	57.5	54.9	54.6	51.8	44.4	36
NOSC ^1^	0	0	1	1	2	2
NOMS ^2^	88	102	102	99	96	92
Percentage of air quality of different grades (%)						
Excellent (0–50) ^3^	13	14	23	23	26	33
Good (51–100) ^3^	33	36	36	42	50	47
Lightly Polluted (101–150) ^3^	27	25	24	20	14	12
Moderately Polluted (151–200) ^3^	12	12	8	7	4	5
Heavily Polluted (201–300) ^3^	13	12	7	6	5	3
Severely Polluted (>300) ^3^	2	1	2	2	1	0

^1^ Number of reaching standard cities; ^2^ Number of PM_2.5_ monitoring stations; ^3^ China’s Ambient air quality standards (AQI).

**Table 2:** Global Moran’s index of PM_2.5_ in BTH from 2013–2018.

Year	2013	2014	2015	2016	2017	2018
Moran’s I	0.4	0.7	0.8	0.7	0.7	0.5

**Table 3:** Geographic detector results for PM_2.5_ for BTH from 2013–2018.

Detection Indices (*X*)	2013	2014	2015	2016	2017
Sown Area of Farm Crops (*X*_1_)	0.6	0.6	0.5	0.4	0.5
Urban Green Area (*X*_2_)	0.8	0.4	0.6	0.8	0.7
Gross Domestic Product (*X*_3_)	0.2	0.2	0.3	0.2	0.4
Gross Domestic Product of Secondary Industry (*X*_4_)	0.4	0.3	0.4	0.4	0.3
Completed Floor Space (*X*_5_)	0.8	0.6	0.7	0.8	0.7
Population Density (*X*_6_)	0.7	0.6	0.7	0.7	0.6
Car Ownership (X_7_)	0.02	0.02	0.02	0.01	0.01
Average Wind Speed (X_8_)	0.3	0.2	0.1	0.1	0.01
Relative Humidity (X_9_)	0.4	0.4	0.4	0.3	0.4
Precipitation (X_10_)	0.5	0.6	0.2	0.01	0.6

## Supplementary Materials

The following are available online at https://www.mdpi.com/1660-4601/16/21/4276/s1: Figure S1: The map of Bohai Rim Economic Circle, Figure S2. The principle of geographical detector, Figure S3. Remote sensing images of cultivated land of the study area in different time, Figure S4. Map of the agricultural information. Chinese Agricultural Natural Regionalization shows in (a); Chinese Land Use Data shows in (b); Chinese farmland ripening data shows in (c), Figure S5. The population density map in BTH region, Table S1. Concentration limit standard of PM_2.5_, Table S2. The proportion of concentration intervals at stations and cities of BTH from 2013–2018, Table S3. The interpretation of detection indices, Table S4. Interactive detection results of PM_2.5_ for BTH from 2013–2017, Table S5. Main legislations and policies on air protection adopted in China, particularly in the BTH region.

## References

[1] Ministry of Environmental Protection of the People’s Republic of China & General Administration of Quality Supervision, Inspection and Quarantine (2012). Ambient Air Quality Standards.

[2] Hsu S.-C., Liu S.C., Jeng W.-L., Lin F.-J., Huang Y.-T., Lung S.-C.C., Liu T.-H., Tu J.-Y. (2005). Variations of Cd/Pb and Zn/Pb ratios in Taipei aerosols reflecting long-range transport or local pollution emissions. Sci. Total Environ..

[3] Deng W., Louie P., Liu W., Bi X., Fu J., Wong M. (2006). Atmospheric levels and cytotoxicity of PAHs and heavy metals in TSP and PM_2.5_ at an electronic waste recycling site in southeast China. Atmos. Environ..

[4] Brunekreef B., Holgate S.T. (2002). Air pollution and health. Lancet.

[5] Xie R., Sabel C.E., Lu X., Zhu W., Kan H., Nielsen C.P., Wang H. (2016). Long-term trend and spatial pattern of PM_2.5_ induced premature mortality in China. Environ. Int..

[6] Laden F., Schwartz J., Speizer F.E., Dockery D.W. (2006). Reduction in Fine Particulate Air Pollution and Mortality: Extended Follow-up of the Harvard Six Cities Study. Am. J. Respir. Crit. Care Med..

[7] Pope C.A., Burnett R.T., Krewski D., Jerrett M., Shi Y., Calle E.E., Thun M.J. (2009). Cardiovascular Mortality and Exposure to Airborne Fine Particulate Matter and Cigarette Smoke: Shape of the Exposure-Response Relationship. Circulation.

[8] Pope C.A., Burnett R.T., Turner M.C., Cohen A., Krewski D., Jerrett M., Gapstur S.M., Thun M.J. (2011). Lung Cancer and Cardiovascular Disease Mortality Associated with Ambient Air Pollution and Cigarette Smoke: Shape of the Exposure–Response Relationships. Environ. Health Perspect..

[9] Polichetti G., Cocco S., Spinali A., Trimarco V., Nunziata A. (2009). Effects of particulate matter (PM_10_, PM_2.5_ and PM_1_) on the cardiovascular system. Toxicology.

[10] WHO (2016). Ambient Air Pollution: A Global Assessment of Exposure and Burden of Disease.

[11] Wu J., Liao X., Peng J. (2015). Simulation and Influencing Factors of Spatial Distribution of PM_2.5_ Concentrations in Chongqing. Environ. Sci..

[12] Peng J., Chen S., Lü H., Liu Y., Wu J. (2016). Spatiotemporal patterns of remotely sensed PM_2.5_ concentration in China from 1999 to 2011. Remote Sens. Environ..

[13] Xue W., Fu F., Wang J. (2014). Numerical study on the characteristics of regional transport of PM_2.5_ in China. China Environ. Sci..

[14] Bai Y., Yang F. (2013). Lessons and Experience Learn from Controlling PM_2.5_ Pollution in the United States. Energy China.

[15] Jiang J., Deng J., Li Z., Li X., Duan L., Hao J. (2014). Sampling Methods for PM_2.5_ from Stationary Sources: A Review. Environ. Sci..

[16] Grover B.D., Eatough N.L., Eatough D.J., Chow J.C., Watson J.G., Ambs J.L., Meyer M.B., Hopke P.K., Al-Horr R., Later D.W. (2006). Measurement of Both Nonvolatile and Semi-Volatile Fractions of Fine Particulate Matter in Fresno, CA. Aerosol Sci. Technol..

[17] Jodeh S., Hasan A.R., Amarah J., Judeh F., Salghi R., Lgaz H. (2017). Indoor and outdoor air quality analysis for the city of Nablus in Palestine: Seasonal trends of PM_10_, PM_5.0_, PM_2.5_, and PM_1.0_ of residential homes. Air Qual. Atmos. Health.

[18] Pant P., Habib G., Marshall J.D., Peltier R.E. (2017). PM_2.5_ exposure in highly polluted cities: A case study from New Delhi, India. Environ. Res..

[19] Duan J., Chen Y., Fang W., Su Z. (2015). Characteristics and Relationship of PM, PM_10_, PM_2.5_ Concentration in a Polluted City in Northern China. Proc. Eng..

[20] Li Q., Li C., Wang Y., Lin C., Yang D., Li Y. (2013). Retrieval on Mass Concentration of Urban Surface Suspended Paticulate Matter with LIDAR and Satellit Remotesensing. Acta Sci. Nat. Univ. Pekin..

[21] Lin C., Li Y., Yuan Z., Lau A.K., Li C., Fung J.C. (2015). Using satellite remote sensing data to estimate the high-resolution distribution of ground-level PM_2.5_. Remote Sens. Environ..

[22] Zhou L., Zhou C., Yang F., Wang B., Sun D. (2017). Spatio-temporal evolution and the influencing factors of PM_2.5_ in China between 2000 and 2011. Acta Geogr. Sin..

[23] Song C., Wu L., Xie Y., He J., Chen X., Wang T., Lin Y., Jin T., Wang A., Liu Y. (2017). Air pollution in China: Status and spatiotemporal variations. Environ. Pollut..

[24] Zhang Y.-L., Cao F. (2015). Fine particulate matter (PM_2.5_) in China at a city level. Sci. Rep..

[25] Li X., Zhang Q., Zhang Y., Zheng B., Wang K., Chen Y., Wallington T.J., Han W., Shen W., Zhang X. (2015). Source contributions of urban PM_2.5_ in the Beijing–Tianjin–Hebei region: Changes between 2006 and 2013 and relative impacts of emissions and meteorology. Atmos. Environ..

[26] Wang G., Xue J., Zhang J. (2016). Analysis of Spatial-temporal Distribution Characteristics and Main Cause of Air Pollution in Beijing-Tianjin Hebei Region in 2014. Meteorol. Environ. Sci..

[27] Wang Z.-B., Fang C.-L. (2016). Spatial-temporal characteristics and determinants of PM_2.5_ in the Bohai Rim Urban Agglomeration. Chemosphere.

[28] Lv B., Hu Y., Chang H.H., Russell A.G., Cai J., Xu B., Bai Y. (2017). Daily estimation of ground-level PM_2.5_ concentrations at 4 km resolution over Beijing-Tianjin-Hebei by fusing MODIS AOD and ground observations. Sci. Total Environ..

[29] Ma X., Wang J., Yu F., Jia H., Hu Y. (2016). Can MODIS AOD be employed to derive PM_2.5_ in Beijing-Tianjin-Hebei over China?. Atmos. Res..

[30] Zhou L., Wu J., Jia R., Liang N., Zhang F., Ni Y., Liu M. (2016). Investigation of Temporal-Spatial Characteristics and Underlying Risk Factors of PM_2.5_ Pollution in Beijing-Tianjin-Hebei Area. Res. Environ. Sci..

[31] PM_2.5_ Mass Concentration Data. http://www.bjmemc.com.cn/.

[32] PM_2.5_ Mass Concentration Data. http://106.37.208.233:20035/.

[33] Meteorological Data. http://data.cma.cn/.

[34] Beijing Municipal Bureau of Statistics & Survey Office of the National Bureau of Statistics in Beijing (2013). Beijing Regional Statistical Yearbook 2013.

[35] Beijing Municipal Bureau of Statistics & Survey Office of the National Bureau of Statistics in Beijing (2014). Beijing Regional Statistical Yearbook 2014.

[36] Beijing Municipal Bureau of Statistics & Survey Office of the National Bureau of Statistics in Beijing (2015). Beijing Regional Statistical Yearbook 2015.

[37] Beijing Municipal Bureau of Statistics & Survey Office of the National Bureau of Statistics in Beijing (2016). Beijing Regional Statistical Yearbook 2016.

[38] Beijing Municipal Bureau of Statistics & Survey Office of the National Bureau of Statistics in Beijing (2017). Beijing Regional Statistical Yearbook 2017.

[39] Beijing Municipal Bureau of Statistics & Survey Office of the National Bureau of Statistics in Beijing (2018). Beijing Regional Statistical Yearbook 2018.

[40] Tianjin Municipal Bureau of Statistics & Survey Office of the National Bureau of Statistics in Tianjin (2013). Tianjin Statistical Yearbook 2013.

[41] Tianjin Municipal Bureau of Statistics & Survey Office of the National Bureau of Statistics in Tianjin (2014). Tianjin Statistical Yearbook 2014.

[42] Tianjin Municipal Bureau of Statistics & Survey Office of the National Bureau of Statistics in Tianjin (2015). Tianjin Statistical Yearbook 2015.

[43] Tianjin Municipal Bureau of Statistics & Survey Office of the National Bureau of Statistics in Tianjin (2016). Tianjin Statistical Yearbook 2016.

[44] Tianjin Municipal Bureau of Statistics & Survey Office of the National Bureau of Statistics in Tianjin (2017). Tianjin Statistical Yearbook 2017.

[45] Tianjin Municipal Bureau of Statistics & Survey Office of the National Bureau of Statistics in Tianjin (2018). Tianjin Statistical Yearbook 2018.

[46] Hebei Provincial People’s Government (2013). Hebei Economic Yearbook 2013.

[47] Hebei Provincial People’s Government (2014). Hebei Economic Yearbook 2014.

[48] Hebei Provincial People’s Government (2015). Hebei Economic Yearbook 2015.

[49] Hebei Provincial People’s Government (2016). Hebei Economic Yearbook 2016.

[50] Hebei Provincial People’s Government (2017). Hebei Economic Yearbook 2017.

[51] Hebei Provincial People’s Government (2018). Hebei Economic Yearbook 2018.

[52] Chinese Agricultural Natural Zoning Data. http://www.resdc.cn/data.aspx?DATAID=273.

[53] Chinese Land Use Remote Sensing Monitoring Data. http://www.resdc.cn/data.aspx?DATAID=264.

[54] Chinese Farmland Ripening Remote Sensing Monitoring Data. http://www.resdc.cn/data.aspx?DATAID=262.

[55] Chinese Population Spatial Distribution Kilometer Grid Data. http://www.resdc.cn/data.aspx?DATAID=251.

[56] The vector Map and DEM (Digital Elevation Model) data. http://ngcc.sbsm.gov.cn/.

[57] Xiao Y., Tian Y., Xu W., Wu J., Tian L., Liu J. (2017). Spatiotemporal Pattern Changes of Air Quality in China from 2005 to 2015. Ecol. Environ. Sci..

[58] Gao K., Zhou X., Yang Y. (2010). Land use structure and its spatial autocorrelation analysis in the Yangtze River basin. Resour. Environ. Yangtze Basin.

[59] Sangalli L.M., Secchi P., Vantini S., Vitelli V. (2010). k-mean alignment for curve clustering. Comput. Stat. Data Anal..

[60] Wang J., Xu C. (2017). Geodetector: Principle and prospective. Acta Geogr. Sin..

[61] Hu Y., Wang J., Li X., Ren D., Zhu J. (2011). Geographical Detector-Based Risk Assessment of the Under-Five Mortality in the 2008 Wenchuan Earthquake, China. PLoS ONE.

[62] Huang J., Wang J., Bo Y., Xu C., Hu M., Huang D. (2014). Identification of Health Risks of Hand, Foot and Mouth Disease in China Using the Geographical Detector Technique. Int. J. Environ. Res. Public Health.

[63] Liao Y., Zhang Y., He L., Wang J., Liu X., Zhang N., Xu B. (2016). Temporal and Spatial Analysis of Neural Tube Defects and Detection of Geographical Factors in Shanxi Province, China. PLoS ONE.

[64] Wang J., Wang Y., Zhang J., Christakos G., Sun J., Liu X., Lu L., Fu X., Shi Y., Li X. (2013). Spatiotemporal transmission and determinants of typhoid and paratyphoid fever in Hongta District, Yunnan Province, China. PLoS Negl. Trop. Dis..

[65] Wang S., Wang Y., Lin X., Zhang H. (2016). Spatial differentiation patterns and influencing mechanism of housing prices in China: Based on data of 2872 counties. Acta Geogr. Sin..

[66] Liu Y., Yang R. (2012). Spatial characteristics and mechanisms of county level urbanization in China. Acta Geogr. Sin..

[67] Tan J., Zhang P., Lo K., Li J., Liu S. (2016). The Urban Transition Performance of Resource-Based Cities in Northeast China. Sustainability.

[68] Xu Q., Zheng X. (2015). Analysis of influencing mechanism of urban growth using geographical detector. Acta Geod. Cartogr. Sin..

[69] Shen J., Zhang N., Gexigeduren, He B., Liu C.-Y., Li Y., Zhang H.-Y., Chen X.-Y., Lin H. (2015). Construction of a GeogDetector-based model system to indicate the potential occurrence of grasshoppers in Inner Mongolia steppe habitats. Bull. Entomol. Res..

[70] Zhang N., Jing Y.-C., Liu C.-Y., Li Y., Shen J. (2016). A cellular automaton model for grasshopper population dynamics in Inner Mongolia steppe habitats. Ecol. Model..

[71] Du Z., Xu X., Zhang H., Wu Z., Liu Y. (2016). Geographical Detector-Based Identification of the Impact of Major Determinants on Aeolian Desertification Risk. PLoS ONE.

[72] Liang P., Yang X. (2016). Landscape spatial patterns in the Maowusu (Mu Us) Sandy Land, northern China and their impact factors. Catena.

[73] Wang W., Tian J., Zhang Y., Wang Q., Han Y., Cao J. (2019). High time resolution emission characteristics and emission inventory of black carbon in typical crop residues burning in China. J. Earth Environ..

[74] Sun S., Li L., Zhao W., Wang L., Qiu Y., Jiang L., Zhang L. (2019). Industrial pollution emissions based on thermal anomaly remote sensing monitoring: A case study of Southern Hebei urban agglomerations, China. China Environ. Sci..

[75] Gao M., Cao J., Seto E. (2015). A distributed network of low-cost continuous reading sensors to measure spatiotemporal variations of PM_2.5_ in Xi’an, China. Environ. Pollut..

[76] Henderson S.B., Beckerman B., Jerrett M., Brauer M. (2007). Application of Land Use Regression to Estimate Long-Term Concentrations of Traffic-Related Nitrogen Oxides and Fine Particulate Matter. Environ. Sci. Technol..

[77] Charron A., Harrison R.M. (2005). Fine (PM_2.5_) and Coarse (PM_2.5–10_) Particulate Matter on A Heavily Trafficked London Highway: Sources and Processes. Environ. Sci. Technol..

[78] Merbitz H., Buttstädt M., Michael S., Dott W., Schneider C. (2012). GIS-based identification of spatial variables enhancing heat and poor air quality in urban areas. Appl. Geogr..

[79] Fang D., Wei Y., Huang W., Cai T., Zhang Y., Liu Q., Zhang Y. (2016). Characterization and Source Apportionment of Organic Carbon during a Heavy Haze Episode in Beijing in October 2014. Res. Environ. Sci..

[80] Bond T.C., Doherty S.J., Fahey D.W., Forster P.M., Berntsen T., DeAngelo B.J., Flanner M.G., Ghan S., Kärcher B., Koch D. (2013). Bounding the role of black carbon in the climate system: A scientific assessment. J. Geophys. Res. Atmos..

[81] Zhang L., Li L., Jiang L., Zhao W., Lu H., Wang X., Qiu Y. (2019). Spatial and Temporal Distribution Characteristics and Fugitive Dust Emission of Building Sites in Beijing. Environ. Sci..

[82] Massey D., Kulshrestha A., Masih J., Taneja A. (2012). Seasonal trends of PM_10_, PM_5.0_, PM_2.5_ & PM_1.0_ in indoor and outdoor environments of residential homes located in North-Central India. Build. Environ..

[83] She L., Xue Y., Guang J., Che Y., Fan C., Li Y., Xie Y. (2018). Towards a comprehensive view of dust events from multiple satellite and ground measurements: Exemplified by the May 2017 East Asian dust storm. Nat. Hazards Earth Syst. Sci..

[84] Yang X., Zhao W., Xiong Q., Wang L., Zhao W. (2017). Spatio-temporal distribution of PM_2.5_ in Beijing-Tianjin-Hebei (BTH) area in 2016 and its relationship with meteorological factors. Ecol. Environ. Sci..

[85] Wang H., Zhao L. (2017). A joint prevention and control mechanism for air pollution in the Beijing-Tianjin-Hebei region in china based on long-term and massive data mining of pollutant concentration. Atmos. Environ..

[86] Zheng Y., Xue T., Zhang Q., Geng G., Tong D., Li X., He K. (2017). Air quality improvements and health benefits from China’s clean air action since 2013. Environ. Res. Lett..

[87] Zhou T., Sun J., Yu H. (2017). Temporal and Spatial Patterns of China’s Main Air Pollutants: Years 2014 and 2015. Atmosphere.

[88] He J., Gong S., Yu Y., Yu L., Wu L., Mao H., Song C., Zhao S., Liu H., Li X. (2017). Air pollution characteristics and their relation to meteorological conditions during 2014–2015 in major Chinese cities. Environ. Pollut..

[89] Wang Y., Zhang J., Wang L., Hu B., Tang G., Liu Z., Sun Y., Ji D. (2014). Researching Significance, Status and Expectation of Haze in Beijing-Tianjin-Hebei Region. Adv. Earth Sci..

[90] Ma Y., Liu N., Hong Y., Wang Y., Zhang Y. (2012). The impacts on various particle sizes and the air quality caused by a dust weather process in Spring 2011 in Liaoning. Acta Sci. Circumstantiae.

[91] Yin C., Zhu B., Cao Y., Sun J., Wang X., Wang H. (2011). The origin of crop residue burning impact on air quality of Nanjing. China Environ. Sci..

[92] Kaiser J.W., Heil A., Andreae M.O., Benedetti A., Chubarova N., Jones L., Morcrette J.-J., Razinger M., Schultz M.G., Suttie M. (2012). Biomass burning emissions estimated with a global fire assimilation system based on observed fire radiative power. Biogeosciences.

[93] Yadav I.C., Devi N.L., Li J., Syed J.H., Zhang G., Watanabe H. (2017). Biomass burning in Indo-China peninsula and its impacts on regional air quality and global climate change-a review. Environ. Pollut..

[94] Levine J. (1991). Global Biomass Burning: Atmospheric, Climatic, and Biospheric Implications.

[95] Cheng L., Fan M., Chen L., Jiang T., Su L. (2017). Effects on the haze pollution from autumn crop residue burning over the Jing-Jin-Ji Region. China Environ. Sci..

[96] Liu A., Wu Q., Chen Y., Zhao T., Cheng X. (2018). Estimation of dust emissions from bare soil erosion over Beijing plain area. China Environ. Sci..

[97] Hu M., Tang Q., Peng J., Wang E., Wang S., Chai F. (2011). Study on Characterization and Source Apportionment of Atmospheric Particulate Matter in China. Environ. Sustain..

[98] Li S., Chen N., Xu J., Nie L., Meng F., Pan T., Tang W., Zhang Y., Alateng T., Dai Z. (2016). Spatial-temporal characteristics of the PM_2.5_ in China in 2014. Acta Geogr. Sin..

[99] Yan D., Lei Y., Shi Y., Zhu Q., Li L., Zhang Z. (2018). Evolution of the spatiotemporal pattern of PM_2.5_ concentrations in China—A case study from the Beijing-Tianjin-Hebei region. Atmos. Environ..

[100] Wang Z., Fang C., Xu G., Pan Y. (2015). Spatial-temporal characteristics of the PM_2.5_ in China in 2014. Acta Geogr. Sin..

[101] Chen H., Li Q., Wang Z., Sun Y., Mao H., Cheng B. (2018). Utilization of MERSI and MODIS data to monitor PM_2.5_ concentration in Beijing–Tianjin–Hebei and its surrounding areas. J. Remote Sens..

[102] Liu J., Lv W., Hao S., Liu Y., Xu D. (2019). Spatial and Temporal Distribution Characteristics of PM_2.5_ Concentration in Beijing-Tianjin-Hebei Region in 2018. Pract. J. Card. Cereb. Pneumal Vasc. Dis..

[103] Meng X., Zhang X., Hou Y., Li J., Jia G., Ye C., Gong Z. (2018). Characteristics of PM_2.5_ concentration in Beijing-Tianjin-Hebei region from 2013 to 2017. Environ. Monit. China.

[104] Deng Q., Yang K., Luo Y. (2017). Spatiotemporal patterns of PM_2.5_ in the Beijing–Tianjin–Hebei region during 2013–2016. Geol. Ecol. Landsc..

[105] Lei J., Zhou H., Lai Z., Bai L., Chen Z. (2018). Analysis of spatio-temporal characteristic of PM_2.5_ concentrations of Chinese cities: 2015–2017. Acta Sci. Circumstantiae.

