# Supplementary material for: Spatio-Temporal Variation Characteristics of PM2.5 in the Beijing–Tianjin–Hebei Region, China, from 2013 to 2018
Source: Int J Environ Res Public Health. 2019 Nov 4;16(21):4276. doi: 10.3390/ijerph16214276 (PMC6862089; doi:10.3390/ijerph16214276)
Supplement: Supplementary file 1 [file ijerph-16-04276-s001.pdf]

International Journal of
Environmental Research and Public Health

Supplementary Materials:

Spatio-Temporal Variation Characteristics of $PM_{2.5}$ in the Beijing–Tianjin–Hebei Region, China, from 2013 to 2018

Lili Wang, Qiulin Xiong [2], Gaofeng Wu, Atul Gautam, Jianfang Jiang, Shuang Liu, Wenji Zhao * and Hongliang Guan *

Table S1. Concentration limit standard of $PM_{2.5}$.

Average Time	Concentration Limit (μg/m³)	
	Grade I Standard	Grade II Standard
Annual Average	15	35
24-hour Average	35	75
Data Validity Provisions		
a. There are at least 324 daily average concentrations per year.		
b. There are at least 27 daily average concentrations per month.		
c. There are at least 25 daily average concentrations in February.		
d. There are at least 20 hours of average concentrations or sampling times per day.		

Table S2. The proportion of concentration intervals at stations and cities in the BTH region from 2013–2018.

Statistic	2013	2014	2015	2016	2017	2018
Proportion of stations at (0,35)	0	0	3%	5%	7%	9%
Proportion of stations at (35,75)	20%	15%	34%	47%	66%	88%
Proportion of stations at (75,150)	80%	85%	63%	47%	23%	3%
Proportion of cities at (0,35)	0	0	1	1	2	2
Proportion of cities at (35,75)	3	3	4	7	6	11
Proportion of cities at (75,150)	10	10	8	5	5	0
The numbers in "(,)" represent the $PM_{2.5}$ mass concentrations in μg/m³.						

Table S3. The interpretation of detection indices.

Detection Indices (X)	The Interpretation of Detection Indices
Sown Area of Farm /hectare	The sown area of farm crops refers to the area actually sown or transplanted with crops. Whether planted on arable land or on non-arable land, they are included in the sown area of farm crops.
Urban green area /hectare	Urban green area refers to the area of land that can be used for greening, excluding roof greening, vertical greening, and land covered with soil of less than 2 m depth. This includes six categories: public green spaces, residential green spaces, unit attached green spaces, protective green spaces, production green spaces, and scenic forest lands.
Gross Domestic Product /100 million yuan	Gross Domestic Product (GDP) refers to the final results of the production activities of all resident units over a certain period of time. GDP is equal to the sum of the added value of each industry.
Gross Domestic Product of Secondary Industry/100 million yuan	The sum of the gross national product of the secondary industry. The secondary industry refers to industries (including extractive industries, manufacturing, tap water, electricity, steam, hot water, gas) and construction.

Population Density /(person/sq.km)	Population density is the number of people per unit area of land.
Car Ownership /unit	Car ownership refers to the number of vehicles owned by the people living in an area, which generally refers to locally registered vehicles.
Completed Floor Space /sq. m	The sum of the planar areas of all floors from one outer wall line of a house to another.
Average Wind Speed /(0.1 m/s)	The average wind speed refers to the average value per 3 seconds, 2 minutes, and 10 minutes over a specified period of time. The average values for 2 minutes were used in this study.
Precipitation /0.1 mm	In China, the National Meteorological Administration (NMA) ground observation standard stipulates that precipitation refers only to vertical precipitation; horizontal precipitation is not treated as precipitation. Precipitation does not necessarily involve actual precipitation; only effective precipitation involves actual precipitation.
Relative Humidity /1%	Relative humidity represents the ratio of the absolute humidity in the air to the saturated absolute humidity at the same temperature and pressure.

Table S4. Interactive detection results of $PM_{2.5}$ for BTH from 2013–2017.

**Interactive Detection Results of $PM_{2.5}$ for BTH in 2013**										
**Detection indices (X)**	X_1	X_2	X_3	X_4	X_5	X_6	X_7	X_8	X_9	X_{10}
Sown Area of Farm Crops(X_1)	0.6									
Urban Green Area(X_2)	**0.9**	0.8								
Gross Domestic Product(X_3)	**0.8**	**0.9**	0.2							
Gross Domestic Product of Secondary Industry(X_4)	**0.8**	**0.9**	**0.5**	0.4						
Completed Floor Space(X_5)	**0.9**	**0.9**	**0.8**	**0.9**	0.8					
Population Density(X_6)	**0.9**	**0.9**	**0.8**	**0.9**	0.8	0.7				
Car Ownership(X_7)	**0.7**	**0.9**	0.2	**0.5**	0.8	0.7	0.02			
Average Wind Speed(X_8)	**0.8**	**0.9**	**0.6**	**0.7**	0.8	**0.8**	**0.4**	0.3		
Relative Humidity(X_9)	**0.9**	**0.9**	**0.6**	**0.7**	0.8	0.7	**0.4**	**0.6**	0.4	
Precipitation(X_{10})	**0.9**	**0.9**	**0.6**	**0.7**	**0.9**	**0.9**	**0.5**	**0.7**	**0.6**	0.5
Interactive detection results of $PM_{2.5}$ for BTH in 2014										
Detection indices (X)	X_1	X_2	X_3	X_4	X_5	X_6	X_7	X_8	X_9	X_{10}
Sown Area of Farm Crops(X_1)	0.5									
Urban Green Area(X_2)	**0.8**	0.4								
Gross Domestic Product(X_3)	**0.8**	**0.5**	0.2							
Gross Domestic Product of Secondary Industry(X_4)	**0.8**	**0.5**	**0.4**	0.3						
Completed Floor Space(X_5)	**0.8**	**0.8**	**0.7**	**0.8**	0.6					
Population Density(X_6)	**0.8**	**0.7**	**0.7**	**0.6**	**0.7**	0.6				
Car Ownership(X_7)	**0.6**	**0.5**	0.2	**0.4**	**0.7**	0.6	0.02			
Average Wind Speed(X_8)	**0.7**	**0.7**	**0.4**	**0.5**	**0.7**	0.6	**0.3**	0.2		
Relative Humidity(X_9)	**0.7**	**0.8**	**0.6**	**0.7**	**0.7**	0.6	**0.5**	**0.6**	0.4	
Precipitation(X_{10})	**0.8**	**0.8**	**0.8**	**0.7**	**0.8**	**0.8**	**0.7**	**0.7**	**0.8**	0.6
Interactive detection results of $PM_{2.5}$ for BTH in 2015										
Detection indices (X)	X_1	X_2	X_3	X_4	X_5	X_6	X_7	X_8	X_9	X_{10}
Sown Area of Farm Crops(X_1)	0.5									
Urban Green Area(X_2)	**0.9**	0.6								
Gross Domestic Product(X_3)	**0.8**	0.6	0.3							
Gross Domestic Product of Secondary Industry(X_4)	**0.7**	**0.9**	**0.7**	0.4						

Completed Floor Space(X_5)	**0.9**	**0.9**	**0.9**	**0.8**	0.7					
Population Density(X_6)	**0.8**	**0.9**	**0.7**	**0.7**	**0.8**	0.7				
Car Ownership(X_7)	**0.6**	0.6	0.3	0.4	**0.8**	0.7	0.02			
Average Wind Speed(X_8)	**0.7**	**0.7**	**0.4**	**0.6**	**0.8**	**0.8**	**0.1**	0.01		
Relative Humidity(X_9)	**0.8**	**0.9**	**0.8**	**0.6**	**0.8**	**0.8**	**0.4**	**0.5**	0.4	
Precipitation(X_{10})	**0.7**	**0.8**	**0.7**	**0.8**	**0.9**	**0.9**	**0.3**	**0.5**	**0.8**	0.2
Interactive detection results of $PM_{2.5}$ for BTH in 2016										
Detection indices (X)	X_1	X_2	X_3	X_4	X_5	X_6	X_7	X_8	X_9	X_{10}
Sown Area of Farm Crops(X_1)	0.4									
Urban Green Area(X_2)	**0.8**	0.8								
Gross Domestic Product(X_3)	**0.8**	0.8	0.2							
Gross Domestic Product of Secondary Industry(X_4)	**0.7**	0.8	**0.7**	0.4						
Completed Floor Space(X_5)	**0.8**	**0.9**	**0.9**	**0.8**	0.8					
Population Density(X_6)	**0.8**	0.8	**0.8**	**0.8**	0.8	0.7				
Car Ownership(X_7)	0.4	0.8	0.2	0.4	0.8	0.7	0.01			
Average Wind Speed(X_8)	**0.5**	**0.9**	**0.4**	**0.5**	0.8	0.7	**0.1**	0.1		
Relative Humidity(X_9)	**0.7**	**0.9**	**0.6**	**0.7**	0.8	0.7	**0.4**	**0.5**	0.3	
Precipitation(X_{10})	**0.5**	**0.9**	**0.4**	**0.6**	0.8	**0.8**	**0.03**	**0.6**	**0.5**	0.01
Interactive detection results of $PM_{2.5}$ for BTH in 2017										
Detection indices (X)	X_1	X_2	X_3	X_4	X_5	X_6	X_7	X_8	X_9	X_{10}
Sown Area of Farm Crops(X_1)	0.5									
Urban Green Area(X_2)	**0.8**	0.7								
Gross Domestic Product(X_3)	**0.6**	**0.8**	0.4							
Gross Domestic Product of Secondary Industry(X_4)	**0.7**	**0.8**	0.4	0.3						
Completed Floor Space(X_5)	**0.8**	**0.8**	**0.8**	**0.7**	0.7					
Population Density(X_6)	**0.8**	**0.8**	**0.7**	**0.8**	**0.8**	0.6				
Car Ownership(X_7)	0.5	0.7	0.4	**0.4**	0.7	0.6	0.01			
Average Wind Speed(X_8)	**0.6**	**0.8**	0.4	**0.4**	0.7	0.6	**0.02**	0.01		
Relative Humidity(X_9)	**0.7**	**0.8**	**0.6**	**0.7**	0.7	0.6	**0.4**	**0.5**	0.4	
Precipitation(X_{10})	**0.7**	**0.8**	**0.8**	**0.8**	**0.8**	**0.8**	**0.6**	**0.6**	**0.8**	0.6

Table S5. Main legislation and policies on air protection adopted in China, particularly in the BTH region.

Publishing Agency	Release time	Name of Legislation or Policy	The Main Content
State Council of China	September 10, 2013	Air pollution prevention and control action plan	Strengthen the comprehensive control of air pollution in industrial enterprises to reduce multi-pollutant emissions; control the total coal consumption, increase the supply of natural gas, increase the intensity of non-fossil energy use and other measures to replace coal combustion; play the role of market mechanism regulation, improve environmental and economic policies
Beijing Municipal Government	September 13, 2013	Beijing Clean Air Action Plan for 2013-2017	Urban functions and spatial layout should be optimized, the population size should be reasonably controlled, and motor vehicle ownership should be strictly controlled. Enhancing the guarantee of clean energy supply, realizing the gasification of electric power production, promoting "coal reduction

			and coal replacement" in urban-rural fringe and rural areas, promoting coal reduction in suburban counties, renovating small-scale pollution enterprises, building ecological industrial parks and promoting clean production.
Beijing Municipal Government	January 22, 2014	Regulations on the Prevention and Control of Air Pollution in Beijing	Establish and improve the working mechanism of government-led, regional linkage, unit governance, participation of the whole people and social supervision; increase the coverage of greening, expand the area of water area, and carry out research on meteorological conditions of atmospheric pollution; prohibit the construction and expansion of facilities burning coal, heavy oil and residue; and implement clean energy transformation of coal-fired heating facilities in remote suburbs within the prescribed time limit.
Standing Committee of the National People's Congress	January 1, 2015	Environmental Protection Law of the People's Republic of China	People's governments at all levels should strengthen publicity and popularization of environmental protection, encourage grass-roots mass self-governing organizations, social organizations, and environmental protection volunteers to carry out publicity on environmental protection laws and regulations and environmental protection knowledge and create a good atmosphere for protecting the environment. People's governments at all levels should strengthen the protection of the agricultural environment, strengthen the monitoring and early warning of agricultural pollution sources, coordinate the relevant departments to take measures to prevent and control soil pollution and ecological imbalances such as land desertification, salinization, barrenness, rocky desertification, vegetation destruction, soil erosion and extinction of provenances.
Tianjin Municipal Government	January 30, 2015	Regulations on Prevention and Control of Air Pollution in Tianjin	Carry out meticulous grid management of air pollution prevention and control, and prohibit the use of high-polluting fuels such as coal and heavy oil, residue and petroleum coke in new, rebuilt and expanded projects in high-polluting fuel no-combustion zones. It is forbidden to incinerate defoliated leaves, straws and withered grass in the open air to produce smoke and dust pollution. Construction projects, house demolition projects, and other construction sites should adopt measures to prevent dust pollution, such as setting up the enclosure, sheathing, road hardening, spraying and so on.

Standing Committee of the Twelfth National People's Congress	August 29, 2015	Law of the People's Republic of China on the prevention and control of air pollution（2015 revision）	Strengthen the comprehensive prevention and control of atmospheric pollution, such as coal combustion, industry, motor vehicles, ships, dust and agriculture, promote joint prevention and control of regional atmospheric pollution, and implement coordinated control of atmospheric pollutants such as particulate matter, sulfur dioxide, nitrogen oxides, volatile organic compounds, ammonia and greenhouse gases. Adjust the energy structure, promote the production and use of clean energy; optimize the use of coal, promote clean and efficient use of coal, gradually reduce the proportion of coal in primary energy consumption, reduce the emission of air pollutants in the process of coal production, use and conversion.
Hebei Municipal Government	January 13, 2016	Regulations on Prevention and Control of Air Pollution in Hebei Province	No new facilities for burning highly polluting fuels such as coal, heavy oil and residue shall be built in the no-combustion zone. The existing facilities for burning highly polluting fuels should be converted to clean energy within a time limit. High-polluting fuel facilities not replaced by clean energy should be equipped with advanced desulfurization, denitrification and dust removal devices or other measures to control the emission of sulfur dioxide, nitrogen oxides, and smoke and dust. If the standards for the discharge of atmospheric pollutants have not yet been met, the use shall be discontinued.
Ministry of Ecology and Environment of the People’s Republic of China	February 17, 2017	A work plan on air pollution prevention and control in BTH region and surrounding areas in 2017	At the end of October of the same year, the government increased its capacity to eliminate excess production and completed the work of banning illegal "small scattered and dirty" enterprises. At the end of October, the construction of the "forbidden coal area" was completed. Cement and foundry industries continue to implement peak staggering production in an all-round way. The port is prohibited from receiving coal transported by diesel trucks. Open-air incineration of straw, litter, and garbage is forbidden in all respects.
Ministry of Ecology and Environment of the People’s Republic of China	August 21, 2017	Air Pollution Comprehensive Control Action Plan for air pollution prevention and control in the BTH region and surrounding areas (key plan)	Industrial enterprises bulk material implementation of peak transportation; Shijiazhuang, Tangshan, Handan, Anyang heating season steel production capacity limited to 50%; Beijing, Tianjin, Baoding, Langfang, Tangshan, Zhangzhou and Xiongan new area and other urban built-up areas are prohibited from using black smoke and high-emission engineering machinery (including excavators, loaders, graders, pavers, road rollers, forklift trucks). In the key areas such as urban built-up

			areas and surrounding areas, and transportation truck lines, the burning of crop straws and other materials that produce soot is completely prohibited. The municipal built-up areas are prohibited from burning domestic garbage, litter, and weeds.
State Council of China	June 27, 2018	Three-year Action Plan for Winning the Blue Sky Defense War	The key remediation areas are Beijing-Tianjin-Hebei and its surrounding areas, and the Yangtze River Delta region. Optimize the industrial layout, increase the adjustment of regional industrial layout, accelerate the relocation or closure of heavy polluting enterprises in urban built-up areas, and comprehensively carry out the "disintegration" enterprises and cluster comprehensive remediation actions. Carry out comprehensive rectification of coal-fired boilers, accelerate the upgrading of rural "coal to electricity" power grids, and accelerate the development of clean energy and new energy.

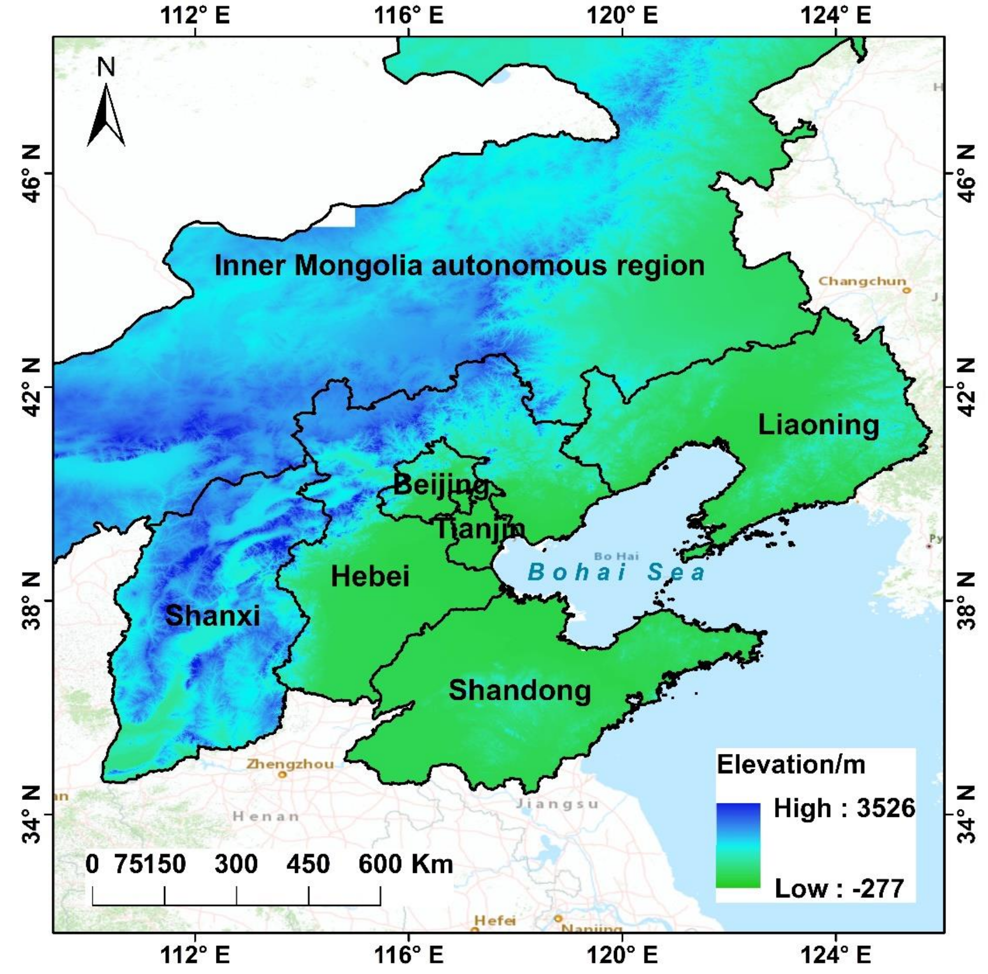

Figure S1. The map of Bohai Rim Economic Circle.

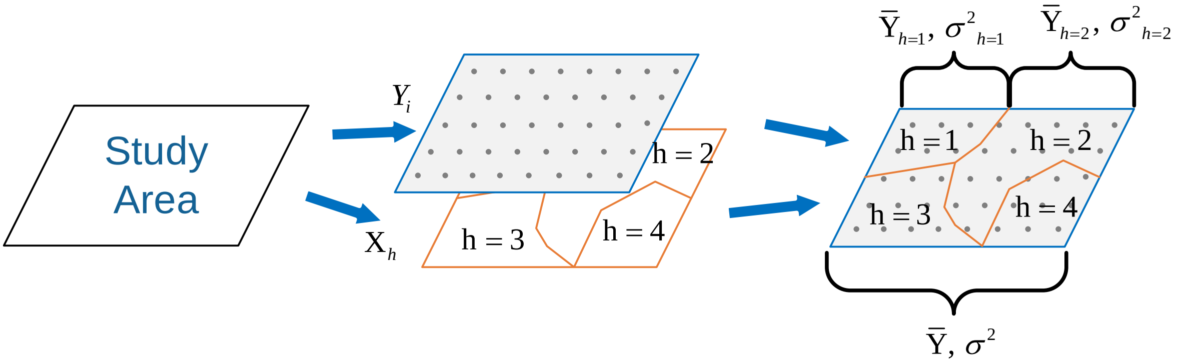

Figure S2. The principle of the geographical detector.

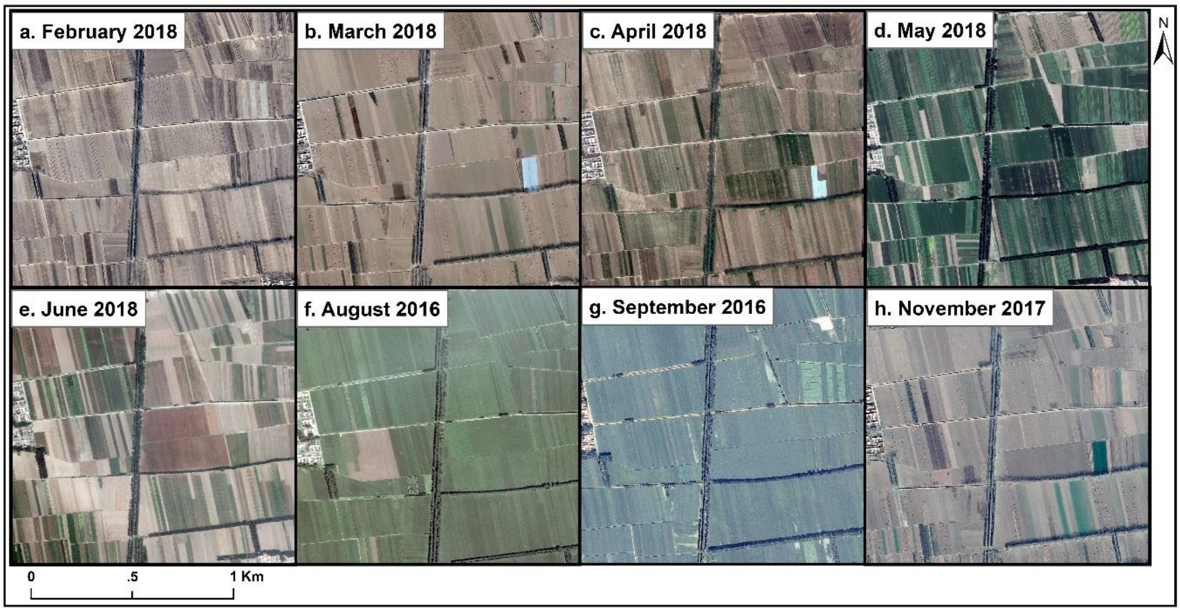

Figure S3. Remote sensing images of cultivated land in the BTH region at different times.

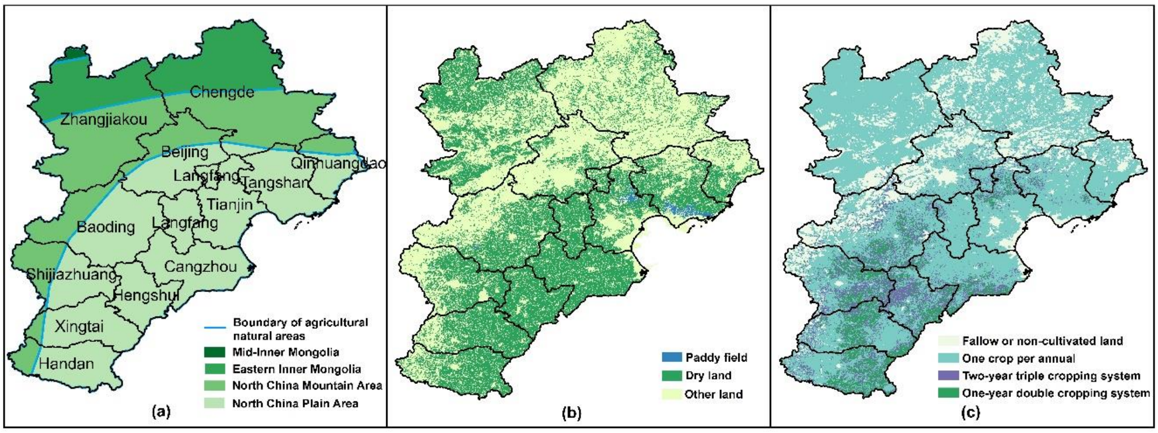

Figure S4. Map of the agricultural information. Chinese Agricultural Natural Regionalization shows in (**a**); Chinese Land Use Data shows in (**b**); Chinese farmland ripening data shows in (**c**).

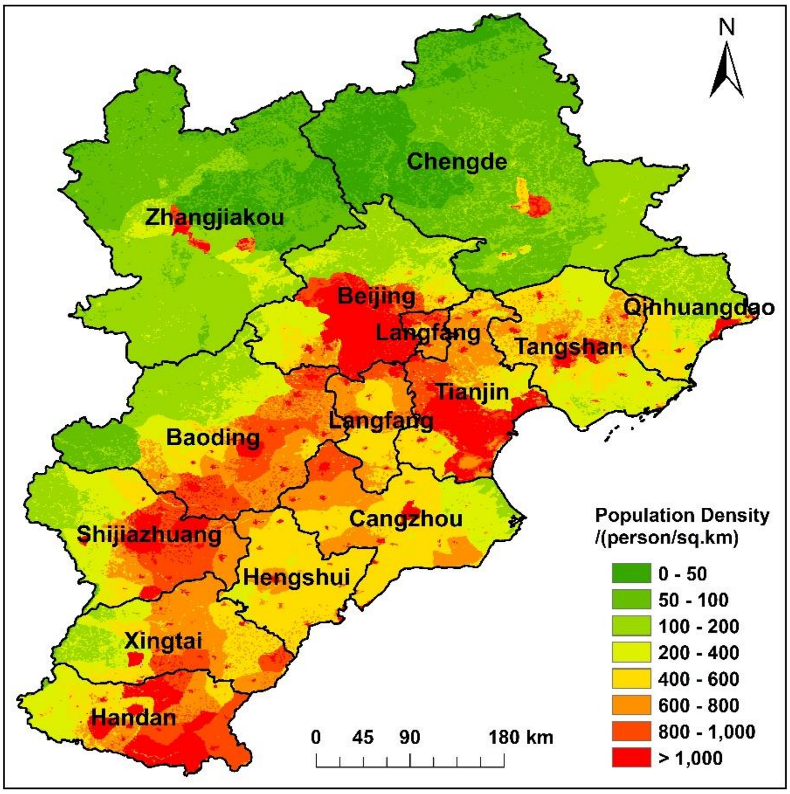

Figure S5. The population density map in BTH region.
